# A screen to identify antifungal antagonists reveals a variety of pharmacotherapies that induce echinocandin tolerance in *Candida albicans*

**DOI:** 10.1128/aac.00484-25

**Published:** 2025-08-18

**Authors:** Parker Reitler, Christian A. DeJarnette, Ravinder Kumar, Katie M. Tucker, Tracy L. Peters, Nathaniel R. Twarog, Anang A. Shelat, Glen E. Palmer

**Affiliations:** 1 Department of Clinical Pharmacy and Translational Science, College of Pharmacy, University of Tennessee Health Sciences Center 550089, Memphis, Tennessee, USA; 2 Department of Pharmacy and Pharmaceutical Sciences, St. Jude Children's Research Hospital 5417, Memphis, Tennessee, USA; 3 Department of Chemical Biology and Therapeutics, St. Jude Children's Research Hospital 5417, Memphis, Tennessee, USA; University of Iowa, Iowa City, Iowa, USA

**Keywords:** *Candida albicans*, echinocandin, antifungal resistance

## Abstract

Through screening a comprehensive collection of drugs approved for human use, we identified over 20 that oppose the antifungal activity of the echinocandins upon the infectious yeast, *Candida albicans*. More detailed evaluation of five such drugs, including the atypical antipsychotic aripiprazole and the tyrosine kinase inhibitor ponatinib, indicated they promote *C. albicans* survival following exposure to the echinocandin antifungals. The activity of the five selected antagonists was dependent upon the Mkc1p mitogen-activated protein kinase pathway; however, ponatinib was paradoxically shown to suppress phosphorylation and therefore activation of Mkc1p itself. Components of several other signaling pathways are also required, including those of calcineurin and casein kinase-2, suggesting the observed antagonism required much of the cell wall stress responses previously described for *C. albicans*. Transcriptome analysis revealed that the antagonists stimulated the expression of genes involved in xenobiotic and antifungal resistance and suppressed the expression of genes associated with hyphal growth. Thus, the echinocandin antagonistic drugs modulate *C. albicans* physiology in ways that could impact its pathogenicity and/or response to therapeutic intervention. Finally, a mutant lacking the Efg1p transcription factor, which has a central role in the activation of *C. albicans* hyphal growth, was found to have intrinsically high levels of echinocandin tolerance, suggesting a link between modulation of morphogenesis-related signaling and echinocandin tolerance.

## INTRODUCTION

The Centers for Disease Control and Prevention estimates the direct healthcare costs of treating fungal disease to be over $7 billion each year in the U.S.A. alone (1). Infections involving fungal invasion to the deeper organs are of greatest concern and predominantly occur in immunocompromised individuals. Several *Candida* spp. account for more than 75% of disseminated fungal infections in the U.S.A. (2, 3), with attributable mortality rates of 35%–75% (4–6). They are also a common cause of mucosal diseases, including oral and vaginal thrush, with *Candida albicans* being the most prevalent and virulent (7). The echinocandins are the first-line therapy for invasive *Candida* infections and disrupt fungal cell wall synthesis through inhibiting β (1,3)-D-glucan synthase (8). However, nearly a third of patients with invasive candidiasis fail to respond to treatment with an echinocandin (9–12), and outcomes are significantly worse for neutropenic patients (13). The incidence of echinocandin resistance is <0.5% for *C. albicans* (14, 15); thus, genetically encoded antifungal resistance does not account for the majority of treatment failures (16–19). Several host-related factors have been proposed to account for the discordance between *in vitro* susceptibility tests and patient outcomes (18). Certainly, rapid diagnosis and administration of an appropriate antifungal agent can reduce patient mortality (20, 21). The severity of patient immunosuppression may also impact the response to antifungal therapy (19, 22). Either way, many treatment failures remain unexplained. Given the limited therapeutic options available, it is critical to identify and address the causes of therapeutic failure to improve patient outcomes.

As natural inhabitants of the human gastrointestinal (GI) and reproductive tracts, several medically important *Candida* spp. are routinely exposed to medications and other pharmacologically active substances consumed by their human host. Given the fundamental conservation of metabolic and signaling modules between eukaryotic genera, it is likely that many drugs designed to modulate human physiology may dysregulate analogous pathways in resident or infecting fungi (23–26). Yet, the influence of most medications upon fungal physiology, pathogenicity, and antifungal susceptibility remains largely uncharacterized. Moreover, it is unclear if such drug-fungus interactions have the potential to influence the incidence or outcomes of mycotic disease. We and others have previously reported that a wide variety of drugs approved for human use can diminish the capacity of the azole antifungals to inhibit the growth of pathogenic *Candida* spp. (27, 28). We also identified a smaller collection that moderates *C. albicans* sensitivity to the echinocandin, caspofungin (27). These interactions are potentially important determinants of clinical outcome, as chemically induced antifungal resistance occurring within a patient receiving an antagonistic medication would not be detected by *in vitro* antifungal susceptibility testing methods. However, the drug collections screened in previous studies only offered partial coverage of the current pharmacopeia. Additionally, the physiological impact of these drug-fungus interactions and their potential to impact interaction with the mammalian host remain unknown. The purpose of this study was to identify medications that alter *C. albicans* sensitivity to the echinocandins and examine the mechanisms by which they act.

## MATERIALS AND METHODS

### Growth conditions

*C. albicans* was grown on yeast extract-peptone-dextrose (YPD) agar plates at 30°C and incubated in YPD liquid medium unless otherwise stated. Select strains were graciously provided either by Dr. Brian M. Peters or Dr. Todd B. Reynolds. Kinase-defective mutants were kindly provided by Dr. Damian Krystan (29).

### Drug stocks

Stock solutions of each antifungal (caspofungin, micafungin, and anidulafungin), as well as each compound (aripiprazole, cinacalcet, haloperidol, netupitant, and ponatinib), were prepared at 10 mM in dimethyl sulfoxide (DMSO) and diluted to required working concentrations. The Food and Drug Administration (FDA) collection was purchased from SelleckChem, with all 2,701 compounds dissolved in the respective solvents listed (either DMSO or water) at 10 mM in 384-well plates. Each was further diluted, and 1 µL volumes were dispensed into the flat-bottomed 384-well polystyrene plates used for the chemical screens.

### Antifungal antagonism screens

Assays were performed similarly as described (28). The wells of the 384-well flat-bottomed assay plates were seeded with 1 µL of the 1 mM stock solutions of each library compound in DMSO or with DMSO alone (control). SC5314 was grown overnight in YPD medium at 30°C, and the cells were harvested by centrifugation, washed twice in phosphate-buffered saline (PBS), and resuspended at ∼6.25 × 10^4^ cells/mL in Roswell Park Memorial Institute (RPMI) 1640 medium buffered to pH 7 with [3-(N-morpholino)propanesulfonic acid] 2% glucose supplemented with the indicated concentration of caspofungin. A 39 µL volume of the cell-plus-antifungal drug suspension was then dispensed into each well of the drugged 384-well plates to achieve approximately 1,000 cells/well. Control wells had antifungal drug but no test/library compound (antifungal alone) or had neither the antifungal nor the library compound (minus drug/untreated control). The final concentration of DMSO was 0.55% in all wells, with each library compound supplied at a final concentration of 5 µM. After 72 hours of incubation at 35°C, growth was measured as OD_600nm_ without shaking. Wells with visible precipitation were excluded from analysis. Growth was compared to the antifungal alone and untreated control and expressed as percent growth. The growth of each drug-treated well was also compared to antifungal-alone wells, and the *Z*-score was calculated (30). “Hits” were identified as compounds with a *Z*-score of ≥3 in two independently run screens.

### Antifungal susceptibility assays

Antifungal susceptibility testing was performed using the broth microdilution method as described in Clinical and Laboratory Standards Institute document M27-A3 (16), with minor modifications. Each echinocandin was diluted in DMSO, resuspended in Roswell Park Memorial Institute medium adjusted to pH 7 (RPMI-pH 7) (2% glucose) at twice the final concentration, and serially diluted. *C. albicans* strains were grown overnight in YPD at 30°C, resuspended at 1 × 10^4^ cells/mL in RPMI-pH 7, and 100 µL was transferred to wells of a round-bottomed 96-well plate containing an equal volume of diluted echinocandin solution. The final concentration of DMSO was 0.5% for all treatments, with drug-free control wells having DMSO alone. Plates were incubated without shaking for 72 hours at 35°C, with plates scanned every 24 hours using an EPSON Perfection v.700 Photo scanner. Experiments were performed in biological duplicate.

### Growth kinetic assays

Growth curve assays were set up in 96-well plates using RPMI-pH 7 (2% glucose). *C. albicans* strains were grown overnight in YPD at 30°C, and the cell density was adjusted to 1 × 10^4^ cells/mL in the appropriate medium for the growth kinetic assays. An aliquot of cell suspension (100 µL) was mixed with an equal volume of twice the desired drug concentration of each antagonist. Cells were then incubated at 35°C inside a BioTek Cytation 5 plate reader with shaking for 48 hours, and OD_600nm_ was read every 30 min. Data were then analyzed via GraphPad Prism software. These assays were repeated in biological duplicate.

### Cell survival and cell damage assays

For cell survival assays, the *C. albicans* parental strain GP1 and strain CAI4 + pKE1 NLUC were grown overnight in YPD at 30°C. Cells were washed and subcultured at 1 × 10^6^ cells/mL in 10 mL of RPMI-pH 7 (2% glucose) supplemented with 0.5% DMSO (vehicle), 0.4 µM, or 1.6 caspofungin alone or in the presence of 5 µM aripiprazole, cinacalcet, haloperidol, netupitant, or 2.5 µM ponatinib. Cells were incubated at 35°C for 6 hours plus or minus drug with shaking. After incubation, cells were spun down at 3,000 revolutions per minute (rpm) for 5 min and washed twice with sterile dH_2_O. Cells were then resuspended in sterile dH_2_O and serially diluted, and 100 µL of cell solutions was plated onto YPD plates. Plates were then incubated for 48 hours at 30°C, and cell viability was measured by counting colony-forming units (CFUs). For cell damage assays, cells were grown under the same conditions, and a portion of supernatant was collected for measuring luciferase activity. Experiments were repeated in biological triplicate.

For cell damage assays, supernatant was collected from the cells grown in the previously listed condition. Luciferase-based assays were performed as previously described (31). Experiments were performed in biological triplicate, and statistical significance was calculated via one-way analysis of variance (ANOVA) test.

### RNA sequencing analysis

SC5314 was grown in YPD medium at 30°C overnight, then subcultured at 1 × 10^6^ cells/mL into 50 mL RPMI-pH 7 medium supplemented with either 5 µM aripiprazole, cinacalcet, haloperidol, netupitant, 2.5 µM ponatinib, or with 0.5% DMSO (vehicle control). Cells were then incubated at 35°C for 6 hours with shaking. Cells were then harvested at 4°C; the supernatant was removed; and cells were frozen at −80°C. Total cellular RNA was extracted using the hot phenol method (32). Novogene provided RNA library preparation and sequencing analysis as a fee-for-service. Messenger RNA was purified from total RNA using poly-T oligo attached magnetic beads. After fragmentation, the first-strand cDNA was synthesized using random hexamer primers, followed by the second-strand cDNA synthesis using either deoxyuridine triphosphate for directional library or deoxythymidine triphosphate for non-directional library. The library was checked with Qubit and real-time PCR for quantification, and a bioanalyzer was used for size distribution detection. Libraries were then pooled and sequenced on Illumina platforms. Genes were mapped to the SC5314 haploid genome assembly 22. Drug-responsive genes were identified as those significantly upregulated or downregulated compared to their respective vehicle controls, with significant gene expression identified as either >1 or <−1 log fold (*P* value of <0.05, adjusted *P* value of <0.05 to account for false discovery rate). Samples were prepared in independent biological duplicate, and log_2_ fold was converted to fold change and averaged, and the average value was converted to log_2_ fold for the term “AVG log_2_ fold.”

SC5314 strain was inoculated in YPD and incubated overnight at 30°C. The next day, SC5314 was subcultured into 75 mL of pre-warmed RPMI-pH 7 (2 % glucose) supplemented with vehicle, 5 µM of aripiprazole, cinacalcet, haloperidol, netupitant, and 2.5 µM ponatinib alone or in combination with caspofungin. SC5314 was washed with PBS and diluted to an OD_600nm_ of 0.25 in RPMI-pH 7 medium using a spectrophotometer (BioPhotometer from Eppendorf). Flasks were incubated at 35°C with 250 rpm with shaking. After 2 hours of growth, cells were harvested at 4,000 rpm for 30 min, and the supernatant was removed. The cells were resuspended in PBS, transferred into a 1.5 mL screw-capped tube, and harvested at 13,000 rpm for 10 min. Tubes were immediately stored at −80°C.

Cell were removed from −80°C, thawed on ice, and then resuspended in 120 mL buffer containing (300 mM NaCl, 10 mM Tris, pH 8.0, 0.1% NP-40, and 10% glycerol) and containing 1× protease inhibitor cocktail, phosphatase inhibitor cocktail set II (Millipore Sigma, 1:100 dilution), and 1 mM dithiothreitol (33). Glass beads equivalent to the size of a pellet were added into the tube, and the tube was kept in the bead beater Bullet Blender Gold (Next Advance) operated for five cycles of 1 min each, with 1 min incubations on ice for each cycle. After cell lysis, whole cell extract was collected in a fresh 1.7 mL Eppendorf tube and kept on ice. Protein quantification was performed by Bradford assay (Thermo Fisher Scientific, cat #1856210), and protein concentration was calculated comparing the result to a standard curve.

Thirty micrograms of protein sample was mixed with 4× loading buffer (from Bio-Rad, cat #1610747), and protein samples were heated for 5 min at 100°C in a thermocycler. Protein samples were cooled to room temperature and loaded onto a 10% SDS-PAGE gel. The gel was run at a constant 100 V until the dye front reached the bottom of the gel. Proteins were transferred onto nitrocellulose membrane (0.2 µM from Bio-Rad, cat #162-0112) using wet transfer for 1 hour at a constant voltage of 100 V. After transfer, membranes were rinsed with distilled water and stained with Ponceaus S stain (from Thermo Fisher Scientific, cat #A40000279), and blots were imaged after rinsing with distilled water. Blots were then incubated in blocking buffer in Tris-buffered saline with 0.1% Tween 20 (TBST) detergent, pH 7.5 (blocking grade blocker from Bio-Rad cat #170-6404) (TBST diluted from 10× stock from Bio-Rad cat #170-6435) for 1 hour at room temperature. Blots were then incubated overnight with rabbit antiphospho-p44/42 antibody (from Cell Signaling, cat #4370) diluted to 1:2,000 in TBST with (5% non-fat skimmed milk powder in TBST, pH 7.5) at 4°C with shaking overnight. The next day, primary antibodies were removed, and blots were washed with TBST for 10 min three times. After the final wash, blots were incubated for 1 hour at room temperature with horseradish peroxidase-conjugated goat antirabbit IgG (H + L) (from Invitrogen, cat #31460) diluted to 1:10,000 in TBST with 5% non-fat skimmed milk powder. After incubation, non-bound antibodies were removed, and blots were washed thrice as above. Finally, the signal was detected using ECL (SuperSignal West Femto Luminol/Enhancer Solution and SuperSignal West Femto Stable Peroxide Buffer, Thermo Fisher Scientific), and images were captured using Molecular Imager ChemiDoc XRS+ with Image Lab Software (from Bio-Rad). Experiments were performed in biological quadruplicate, and statistical significance was calculated via one-way ANOVA test.

## RESULTS

### Several drugs approved for human use oppose the antifungal activity of echinocandins upon *C. albicans*

To identify medications that potentially modulate the efficacy of echinocandin therapy, we conducted a simple screen. *C. albicans* (strain SC5314) was suspended in RPMI medium supplemented with approximately 4× the minimum inhibitory concentration (MIC) (0.78 µM) of caspofungin and dispensed into 384-well plates arrayed with a collection of 2,701 pharmacologically active small molecules, most of which are approved for human use by the FDA, the European Medicines Agency, and other approval agencies. Each compound was provided to a final concentration of 5 µM, and control wells contained either no drugs or antifungal (DMSO vehicle growth control), or caspofungin alone. Given the cidal mode of action of the echinocandins, we measured fungal growth in each well as OD_600nm_ after 72 hours of incubation at 35°C. This strategy was intended to identify drugs that promote fungal survival in the presence of caspofungin (i.e., tolerance), as well as any that induce outright resistance (i.e., insensitivity). Compounds were called hits if they restored fungal growth in the presence of caspofungin (*Z*-score ≥3) in each of two independent replicate screens. A total of 22 hits, corresponding to 21 distinct chemical entities, were identified as opposing the antifungal activity of caspofungin upon *C. albicans* (Table 1). This included (i) aripiprazole, an atypical antipsychotic that we had previously reported to diminish the antifungal activity of the azole antifungals upon *C. albicans* (34); (ii) cinacalcet, a calcimimetic used to treat hyperparathyroidism; (iii) the antipsychotic haloperidol; (iv) the antiemetic netupitant; and (v) ponatinib, a multi-targeted tyrosine-kinase inhibitor used to treat leukemia. These five drugs were selected as representative hits for further evaluation, and their activity was compared.

**TABLE 1:** Caspofungin antagonists identified from the SelleckChem FDA collection^*a*^*^,^*^*b*^

AVG % growth^*c*^	AVG *Z*-scores^*c*^ (CAS alone)	Product name	Human target	*C. albicans* homolog (% ID)
43.9	29.9	Ponatinib (AP24534)	Bcr-Abl:FGFR:PDGFR:VEGFR	Approx. 40 kinase targets (26%–35%)
45.1	28.4	Haloperidol	5-Hydroxytryptamine receptor 2C, d(2) dopamine receptor	NA, NA
29.4	15.1	Sanguinarine chloride	PP2C	Cch1p (20%)
22.3	13.5	Cinacalcet HCl	Extracellular calcium-sensing receptor	NA
3.4	13.2	Oxaliplatin	DNA	NA
18.9	11.5	Aripiprazole	5-Hydroxytryptamine receptor 2C, d(2) dopamine receptor	NA, NA
20	11.4	Netupitant	Neuropeptide substance-P receptor	NA
21.5	11.1	Sanguinarine	PP2C	Cch1p (20%)
13.7	9.5	Fesoterodine fumarate	Muscarinic acetylcholine receptor M3	NA
14.1	8.9	Proanthocyanidins	NLRP3-inflammasome among other immune inflammatory response pathways	NA
11.2	7.8	Costunolide	Telomerase	NA
12.3	6.6	Vorapaxar	Proteinase-activated receptor 1	NA
9.5	6.4	Bambuterol HCl	Beta-2 adrenergic receptor	NA
10.1	6.3	Methyldopa	Aromatic-L-amino acid decarboxylase, alpha-2A adrenergic receptor	NA, NA
10.9	6.0	Acarbose	Maltase-glucoamylase, sucrase-isomaltase, pancreatic alpha-amylase	Gca1p (33%–34%), Gca2 (33%–39%), Rot2p (28%)
8.5	5.0	Suprofen	Prostaglandin G/H synthase 1, prostaglandin G/H synthase 2	NA, NA
8.2	5.0	Nicardipine HCl	Voltage-dependent L-type calcium channel subunit alpha-1C, beta-2, alpha-2/delta-1, and subunit-1D	Cch1p (21%)/Syg1p (20%), NA, NA, Cch1p (21%)
7.2	4.8	Biotin (vitamin B7)	Vitamin	NA
6.3	3.9	Iopamidol	Diagnostic imaging agent for X-rays	NA
5.7	3.6	Yangonin	Cannabinoid CB1 receptor	NA
5.4	3.4	Clindamycin alcoholate	Large ribosomal subunit uL1 (*Staphylococcus aureus*)	NA
5.1	3.2	Bethanechol chloride	Muscarinic acetylcholine receptor M3, M5	NA, NA

^*a*^ Wild-type *C. albicans* strain SC5314 was dispensed in RPMI-pH 7 2% glucose supplemented with 4× MIC of CAS into 384-well plates arrayed with the SelleckChem FDA collection to a final concentration of 5 µM. Plates were incubated for 72 hours at 35°C, and growth quantified as OD_600nm_. Growth is expressed relative to untreated (DMSO vehicle alone/minus antifungal) control wells (% growth). Hits were called as compounds restoring growth (Z-score of ≥3 compared to antifungal-alone control) in two independently run experiments.

^*b*^ CAS, caspofungin; NA, non-applicable; PP2C, protein phosphatase 2C.

^*c*^ Average of two independently conducted screens.

**Fig 1:**
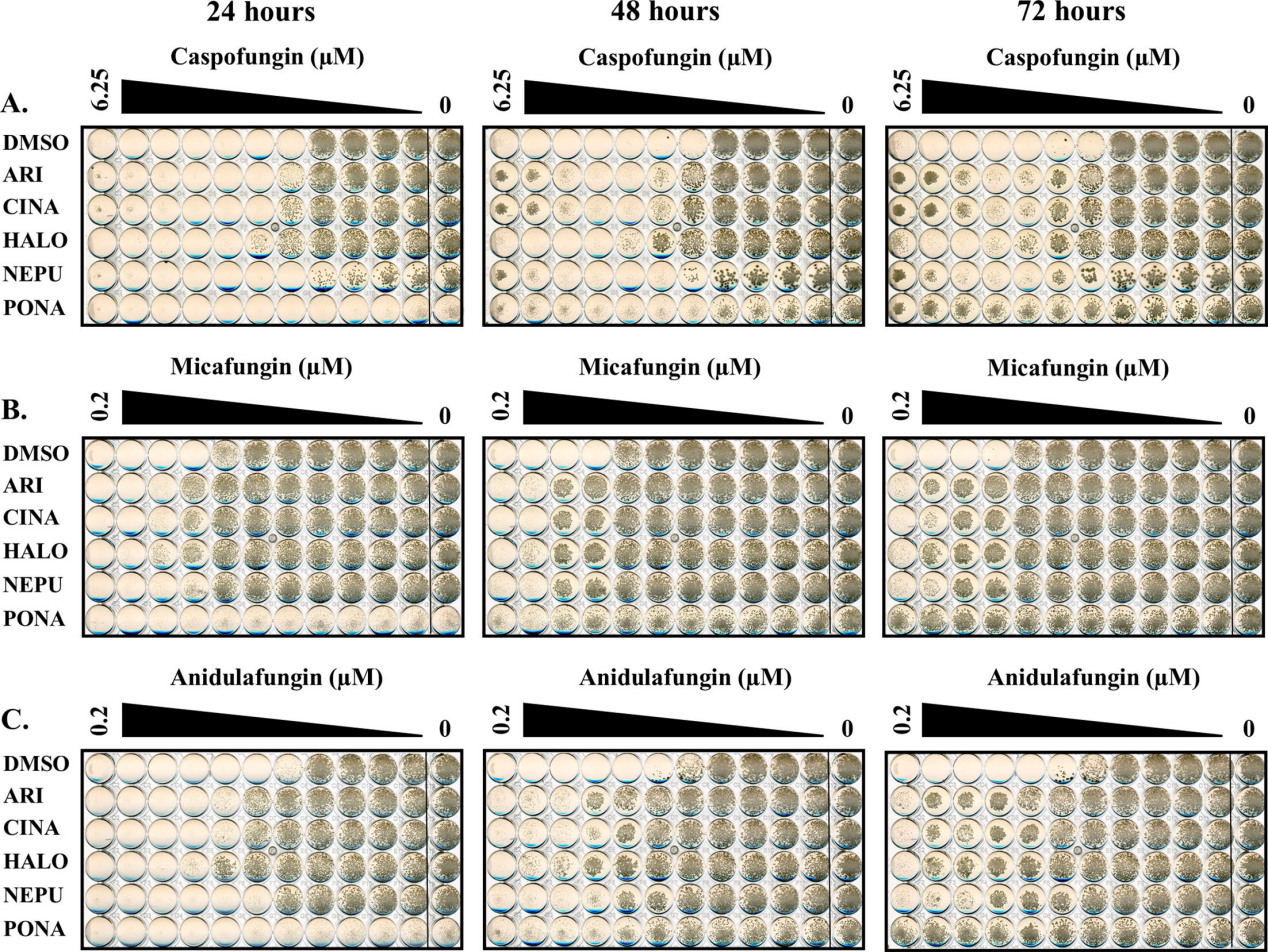
Several medications alter *Candida albicans* tolerance of the echinocandins. Antifungal susceptibility assays were performed with *C. albicans* SC5314 in RPMI-pH 7 (2% glucose) and twofold dilutions of caspofungin (**A**), micafungin (**B**), or anidulafungin (**C**) in the presence of either 5 μM of aripiprazole (ARI), 5 μM cinacalcet (CINA), 5 μM haloperidol (HALO), 5 μM netupitant (NEPU), 2.5 μM ponatinib (PONA), or vehicle (DMSO). Plates were incubated at 35°C and imaged after 24, 48, and 72 hours. Images are representative of assays performed in biological duplicate.

Initial dose-response assays confirmed that all five drugs opposed the antifungal activity of caspofungin, although the degree of protection conferred by each varied. In all cases, their protective effect upon *C. albicans* was progressively more apparent at later time points (i.e., 48 and 72 hours) (Fig. 1A). When read after 24 hours of incubation, caspofungin MICs were comparable for all treatments, but residual growth was apparent at concentrations above the MIC in the presence of all five antagonists. Additionally, with the exception of ponatinib, the other four antagonists appear to preferentially restore *C. albicans* growth at the highest concentrations of caspofungin and therefore act to induce, or enhance, the previously described paradoxical growth observed at high concentrations of this antifungal (35). These data indicate that the antagonists identified do not substantially alter antifungal potency (i.e., MIC) but rather diminish the cidal capacity of caspofungin and promote tolerance. Subsequent checkerboard analysis revealed that many of the antagonists are protective at much lower concentrations than the 5 µM concentrations used in the initial screens (Fig. S1). Given that four of the five antagonists studied lack stand-alone antifungal activity, it was not possible to calculate fractional inhibitory concentration indices (36, 37). However, Bliss independence scores were calculated by assessing the maximum positive Bliss deviation, which corresponds to the most antagonistic interaction with caspofungin (38) (Table 2), and revealed substantial antagonistic effects over specific concentration ranges of each pairwise combination, with up to 65% growth restoration versus the minus antifungal control, by the 72 hour time point.

**TABLE 2:** Bliss independence analysis for antagonist and caspofungin interaction^*a*^

Antagonist	Min Bliss deviation (%)	Max Bliss deviation (%)
Aripiprazole	−1.957 ± 1.287	53.301 ± 8.270
Cincalcet	−1.715 ± 0.452	59.623 ± 18.986
Haloperidol	−0.836 ± 0.827	65.886 ± 3.649
Netupitant	−2.377 ± 2.595	14.753 ± 9.119
Ponatinib	−0.943 ± 1.515	24.539 ± 14.352

^*a*^ Checkerboard assays were performed with caspofungin and each of the five antagonists with *C. albicans* SC5314 in RPMI-pH 7 (2% glucose). Plates were incubated for 72 hours (at 35°C); growth was quantified as OD_600nm_ and expressed relative to the minus drug/vehicle control wells (% growth). Bliss volumes were calculated for each combination of antagonist and caspofungin as the observed deviation from the expected growth, i.e., growth expected based on the combined activity of each individual agent at the same concentration. The minimum (min) and maximum (max) deviations observed for each drug combination are reported for the average ± standard deviation of three independently conducted checkerboard assays.

**Fig 2:**
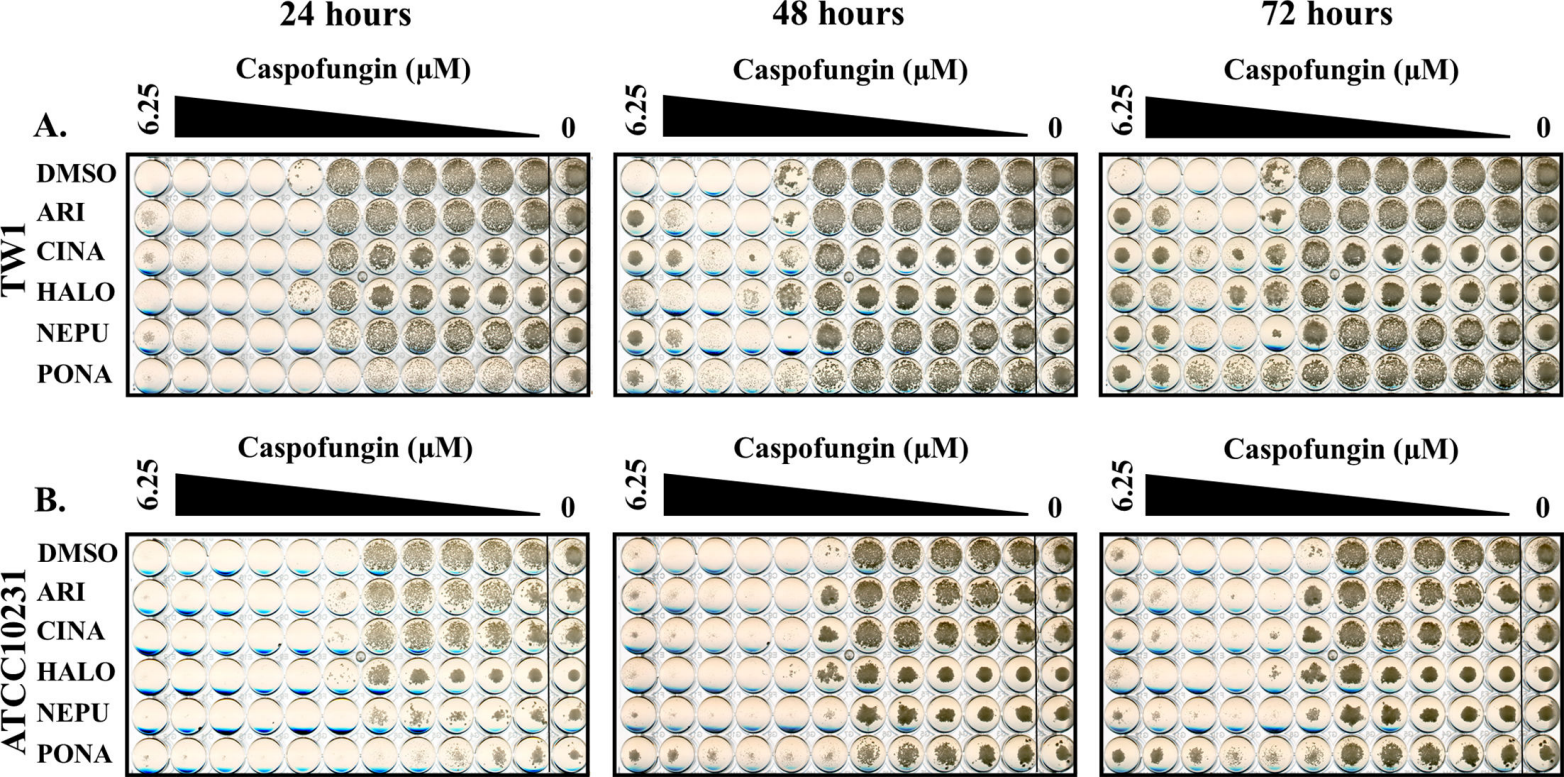
Echinocandin antagonist activity is not strain specific. Caspofungin susceptibility assays were performed with *C. albicans* strain TW1 (**A**) or American Type Culture Collection 10231 (**B**) in RPMI-pH 7 (2% glucose) supplemented with 5 μM aripiprazole (ARI), 5 μM cinacalcet (CINA), 5 μM haloperidol (HALO), 5 μM netupitant (NEPU), 2.5 μM ponatinib (PONA), or vehicle (DMSO). Plates were incubated at 35°C and imaged after 24, 48, and 72 hours. Images are representative of assays performed in biological duplicate.

All five of the selected drugs also enhanced SC5314 growth in the presence of supra-MIC concentrations of micafungin and anidulafungin, the two other echinocandins approved for human use (Fig. 1B and C). All five also oppose the antifungal activity of caspofungin on *C. albicans* strains TW1 (39) and American Type Culture Collection 10231 (Fig. 2A and B). However, in the case of anidulafungin, the antagonists caused an overt increase in MIC rather than enhanced paradoxical growth. Thus, their antagonistic activity is neither strain nor echinocandin specific. Interestingly, at least three of the five antagonists (aripiprazole, cinacalcet, and haloperidol) increased the concentration of caspofungin required to suppress *Candida parapsilosis* growth (Fig. S2A), while both cinacalcet and netupitant also enhanced caspofungin tolerance in *Candida tropicalis* (Fig. S2B), although the effects were less pronounced than those observed for *C. albicans*. Thus, at least a subset of the echinocandin antagonists identified affect the response of multiple medically important *Candida* spp.

### Antagonists diminish cidality and induce echinocandin tolerance

**Fig 3:**
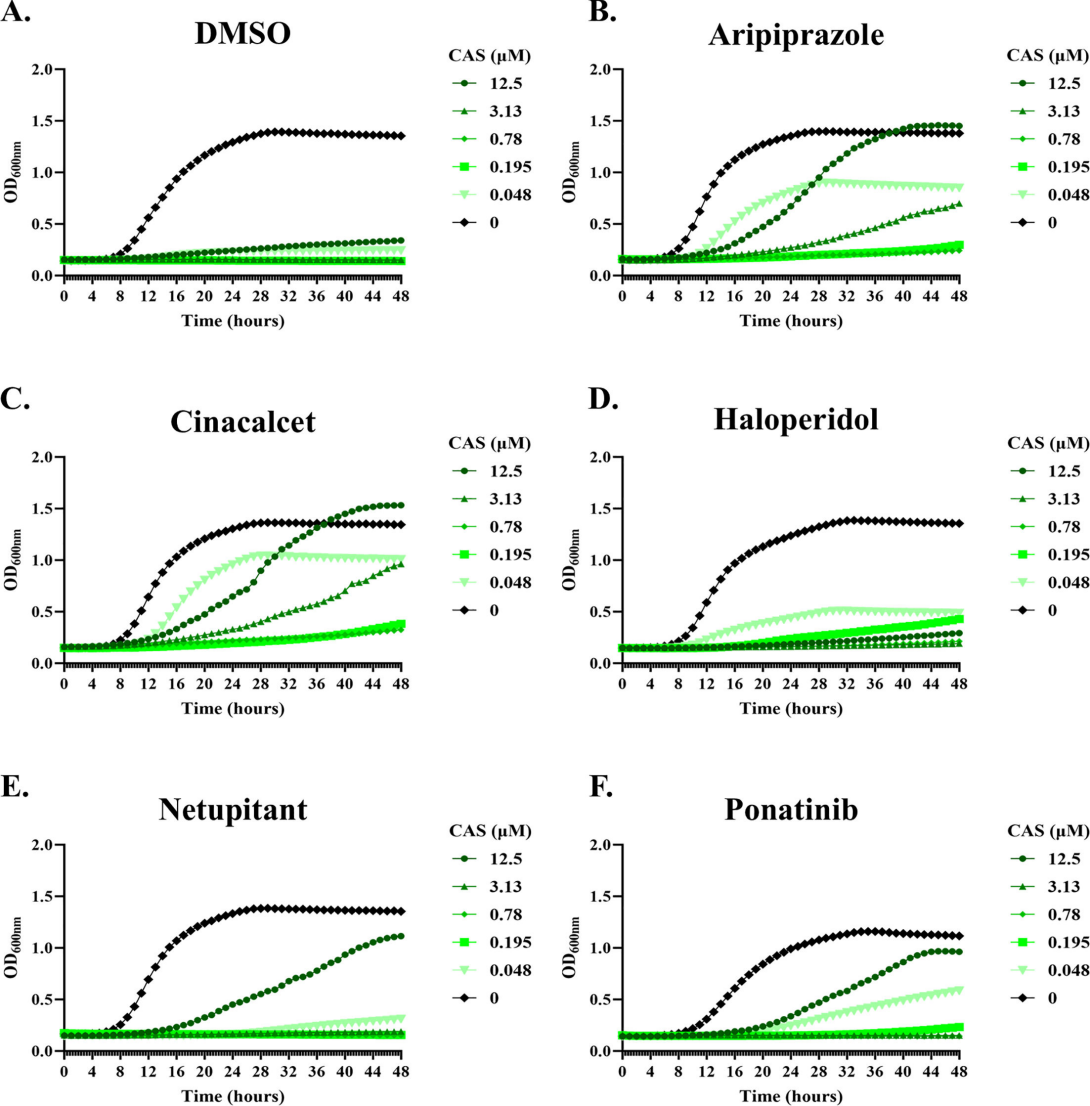
Echinocandin antagonists partially restore *Candida albicans* growth in the presence of caspofungin. SC5314 was seeded RPMI-pH 7 (2% glucose) medium supplemented with vehicle (DMSO, **A**), 5 μM aripiprazole (**B**), 5 μM cinacalcet (**C**), 5 μM haloperidol (**D**), 5 μM netupitant (**E**), or 2.5 μM ponatinib (**F**) and a range of caspofungin (CAS) concentrations. Plates were incubated at 35°C for 48 hours, and OD_600nm_ was measured every 30 min. Results are representative of assays performed in biological duplicate.

To further characterize the mode by which the selected antagonists protect *C. albicans* from the echinocandins, we conducted a more thorough assessment of cell growth, injury, and viability. Growth kinetics in the presence of inhibitory concentrations of caspofungin revealed that the antagonists permit growth but at much slower rates than in the absence of the antifungal (Fig. 3). Furthermore, their effect is more profound at high concentrations of the antifungal. This is consistent with them inducing a form of echinocandin tolerance rather than insensitivity, specifically, enhancing the paradoxical effect.

**Fig 4:**
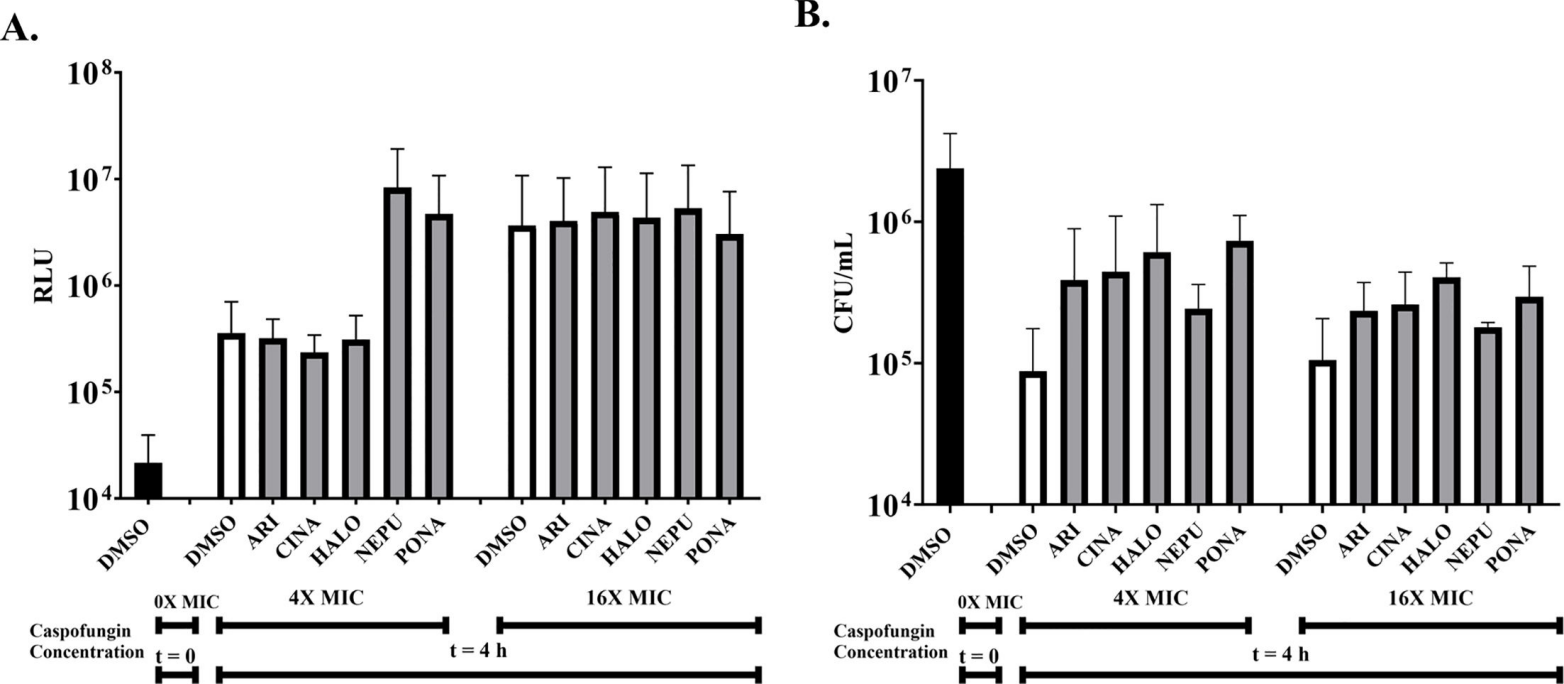
Antagonists do not diminish cell damage but may enhance *Candida albicans* survival of echinocandin exposure. (**A**) A *C. albicans* wild-type strain expressing NanoLuc (NLUC) was subcultured at 5 × 10^6^ cells/mL into RPMI-pH 7 (2% glucose) supplemented with vehicle (0.5% DMSO), 5 μM aripiprazole (ARI), 5 μM cinacalcet (CINA), 5 μM haloperidol (HALO), 5 μM netupitant (NEPU), or 2.5 μM ponatinib (PONA) for 2 hours at 35°C, then caspofungin was added at 0×, 4×, and 16× MIC and further incubated for 4 hours at 35°C. The culture supernatant was collected, and NLUC release was quantified in each sample as relative luminescence units (RLU). (**B**) Cell viability for each sample described in panel A was quantified as colony-forming units (CFUs) after plating samples onto YPD agar. Representative data are shown from three independent experiments. See the main text for further details.

A *C. albicans* strain expressing a small cytoplasmic luciferase (NanoLuc, Promega) (40) was used to compare the overall levels of damage sustained by populations of cells. Following 4 hours of caspofungin exposure at either 4× or 16× the MIC, NanoLuc released into the culture supernatant was approximately 100-fold higher than mock-treated controls (Fig. 4A), indicating substantial levels of cell injury and/or lysis. Comparable levels of NanoLuc were released in the presence of each of the antagonistic drugs, suggesting they do not diminish the damage sustained following caspofungin exposure *per se*. Cell viability was also quantified as CFUs after 4 hours of exposure to 4× MIC of caspofungin. As expected, 4× MIC of caspofungin profoundly reduced *C. albicans* viability (Fig. 4B). The presence of four antagonists improved cell survival in the presence of caspofungin by approximately 2-5-fold, although this difference was not statistically significant due to substantial variation in CFUs between experiments. Nonetheless, these data suggest that the antagonists protect a subpopulation of *C. albicans* cells from the cidal effects of the echinocandins and permit continued proliferation of the surviving cells at a much-reduced rate.

### Echinocandin antagonistic drugs require Mkc1p

**Fig 5:**
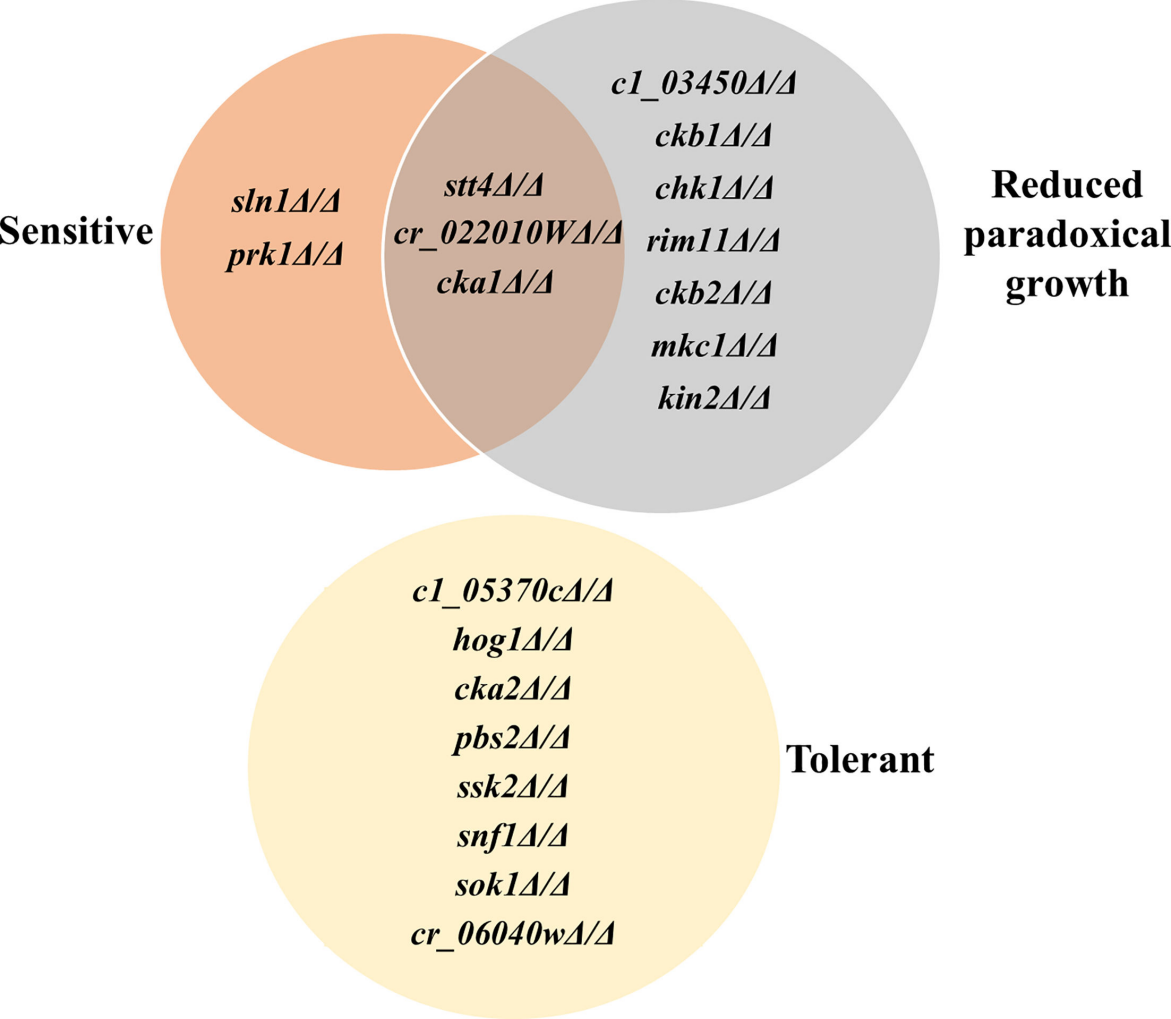
*Candida albicans* deletion mutants with altered echinocandin sensitivity. Ninety-one *C. albicans* gene deletion strains were suspended in RPMI-pH 7 (2% glucose) supplemented with 0, 0.25×, 1.0×, 4.0×, 16.0×, or 64.0× MIC caspofungin. Plates were then incubated at 35°C, and growth was compared visually after 24, 48, and 72 hours. Those that had reduced or no growth compared to the wild-type control at 0.25× MIC were classified as sensitive. Mutants lacking or with reduced paradoxical growth versus the wild-type control were identified at 64× MIC after 72 hours. Finally, tolerant mutants were identified as those with increased growth versus the wild-type in the presence of 1×, 4×, and/or 16× MIC. In each case, the phenotype was observed in each of two independently conducted experiments.

To provide insight into the molecular mechanisms underlying echinocandin antagonism, a collection of 91 deletion mutants lacking genes encoding kinases, or kinase-related products, was probed (29). Initially, the collection was screened to identify gene deletion strains with caspofungin sensitivity. The first screen identified five sensitive mutants with reduced growth compared to wild type after 48 hours in the presence of subinhibitory concentrations of caspofungin (0.25× MIC) (Fig. 5; Table 3). A further eight grew in the presence of supra-growth inhibitory concentrations (4× or 16× MIC), including mutants lacking Hog1p, as well as the Pbs2p MAPKK and Ssk2p MAPKKK, which regulate Hog1p activity (41) (Fig. 5; Table 4). A third screen identified 15 mutants as deficient in paradoxical growth, measured after 72 hours of incubation with 64× MIC of caspofungin (Fig. 5; Table 5). These varied phenotypes (sensitivity as well as the capacity to survive and replicate in the presence of various concentrations of caspofungin) indicate that kinase-mediated signaling is a critical determinant of *C. albicans* cell fate following echinocandin exposure, with both pro-survival and pro-cidal functions evident.

**TABLE 3:** Caspofungin-sensitive *Candida albicans* mutants

Mutant	Pathway^*a*^
*sln1*Δ/Δ	Cell wall biosynthesis
*prk1*Δ/Δ	Putative protein serine/threonine kinase
*cr_02210w*Δ/Δ	Putative serine/threonine protein kinase, involved in the regulation of biofilm formation
*stt4*Δ/Δ	Phosphatidylinositol-4-kinase, forms a complex with Ypp1p and Efr3p that is required for phosphatidylinositol-4-phosphate in plasma membrane
*cka1*Δ/Δ	Putative alpha subunit (catalytic subunit) of protein kinase CK2

^*a*^ As described in *Candida* Genome Database.

**TABLE 4:** Caspofungin-tolerant *Candida albicans* mutants

Mutant	Pathway^*a*^*^,^*^*b*^
*c1_05370c*Δ/Δ	Putative serine/threonine kinase
*hog1*Δ/Δ	MAP kinase of osmotic-, heavy metal-, and core stress response; role in the regulation of response to stress
*cka2*Δ/Δ	Catalytic alpha-subunit of protein kinase CK2, interaction with the calcineurin pathway
*pbs2*Δ/Δ	MAPK kinase, role in osmotic and oxidative stress responses
*ssk2*Δ/Δ	MAP kinase kinase kinase, regulates Hog1 activation and signaling
*snf1*Δ/Δ	Functional homolog of *Saccharomyces cerevisiae* Snf1p, which regulates sugar metabolism
*sok1*Δ/Δ	Protein kinase required for degradation of Nrg1p
*cr_06040w*_aΔ/Δ	Ortholog of *S. cerevisiae* Sat4p, amphotericin B induced, clade-associated gene expression

^*a*^ As described in *Candida* Genome Database.

^*b*^ MAPK, mitogen-activated protein kinase.

**TABLE 5:** *Candida albicans* mutants lacking paradoxical growth with caspofungin

Mutant	Pathway^*a*^
*c1_03450c*Δ/Δ	Protein kinase-related protein, possibly involved in sterol transport between the plasma membrane and ER
*ckb1*Δ/Δ	Regulatory subunit of protein kinase CK2 (casein kinase II), beta subunit
*chk1*Δ/Δ	Histidine kinase; two-component signaling, cell wall synthesis
*rim11*Δ/Δ	Ortholog of *S. cerevisiae* Rim11p, a protein kinase involved in meiosis and sporulation in *S. cerevisiae*
*ckb2*Δ/Δ	Regulatory subunit of protein kinase CK2 (casein kinase II)
*bck1*Δ/Δ	Ortholog of *S. cerevisiae* Bck1p, MAP kinase kinase kinase of the cell integrity pathway
*mkc1*Δ/Δ	MAP kinase; role in biofilm formation, contact-induced invasive filamentation, systemic virulence in mouse, cell wall structure/maintenance
*kin2*Δ/Δ	Protein with similarity to *S. cerevisiae* Kin2p, transcription is positively regulated by Tbf1p
*cr_02210w*Δ/Δ	Putative serine/threonine protein kinase, involved in the regulation of biofilm formation
*stt4*Δ/Δ	Phosphatidylinositol-4-kinase, forms a complex with Ypp1p and Efr3p that is required for phosphatidylinositol-4-phosphate in the plasma membrane
*cka1*Δ/Δ	Putative alpha subunit (catalytic subunit) of protein kinase CK2

^*a*^ As described in *Candida* Genome Database.

**Fig 6:**
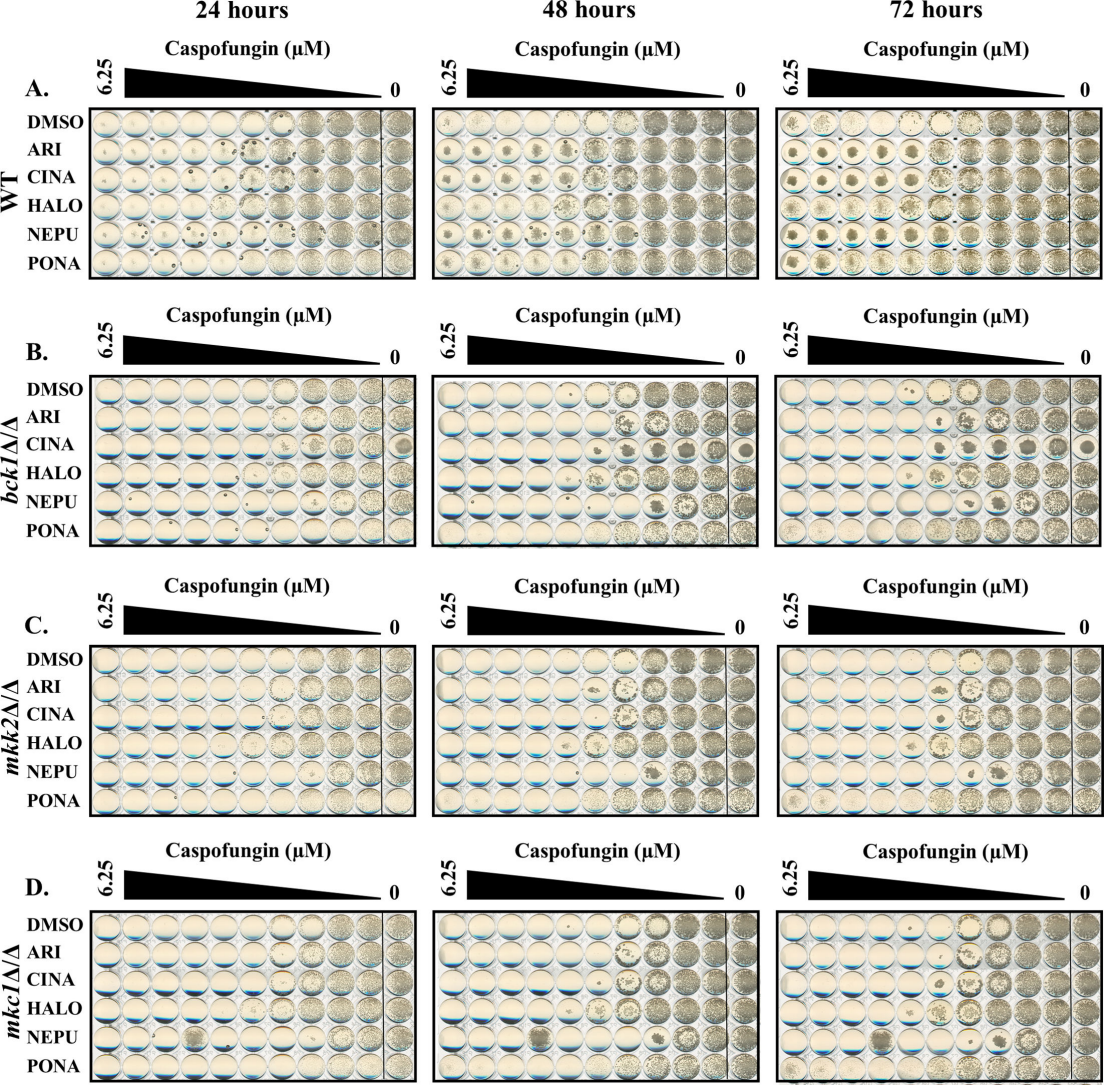
The Mkc1p MAP kinase pathway is required for echinocandin antagonism. Caspofungin susceptibility of wild-type (SN95) (**A**), *bck1*Δ/Δ (**B**), *mkk2*Δ/Δ (**C**), and *mkc1*Δ/Δ (**D**) *C. albicans* strains were compared in RPMI-pH 7 (2% glucose) supplemented with either 5 μM aripiprazole (ARI), 5 μM cinacalcet (CINA), 5 μM haloperidol (HALO), 5 μM netupitant (NEPU), 2.5 μM ponatinib (PONA), or vehicle (DMSO). Plates were incubated at 35°C and imaged after 24, 48, and 72 hours. Images are representative of assays performed in biological duplicate.

**Fig 7:**
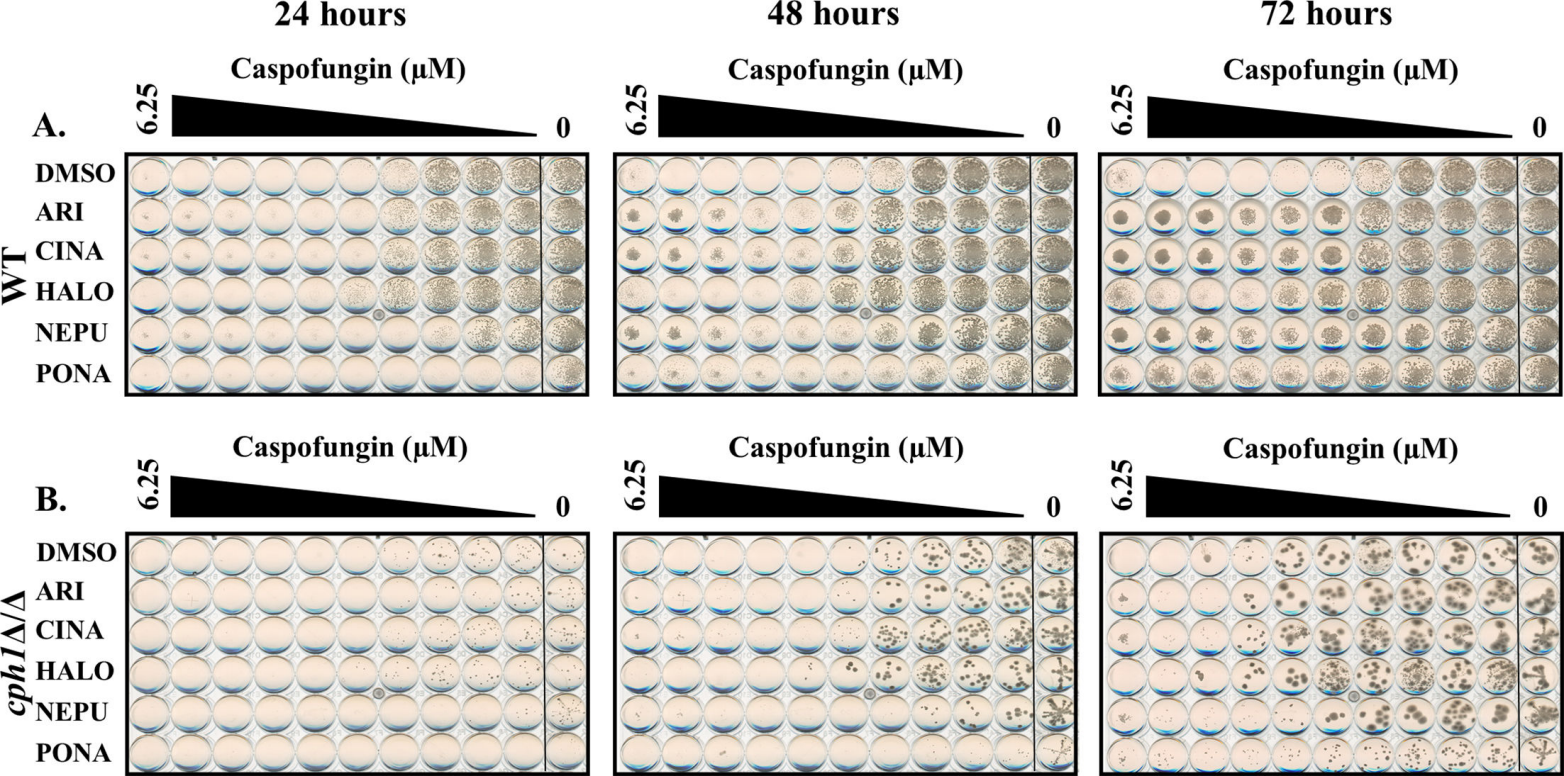
The Cph1p transcription factor is required for echinocandin antagonism in *Candida albicans*. Caspofungin sensitivity of wild-type (SC5314) and (**A**) and *cph1*Δ/Δ (**B**) strains of *C. albicans* was compared in RPMI-pH 7 (2% glucose) supplemented with either 5 μM aripiprazole (ARI), 5 μM cinacalcet (CINA), 5 μM haloperidol (HALO), 5 μM netupitant (NEPU), 2.5 μM ponatinib (PONA), or vehicle (DMSO). Plates were incubated at 35°C and imaged after 24, 48, and 72 hours. Images are representative of assays performed in biological duplicate.

**Fig 8:**
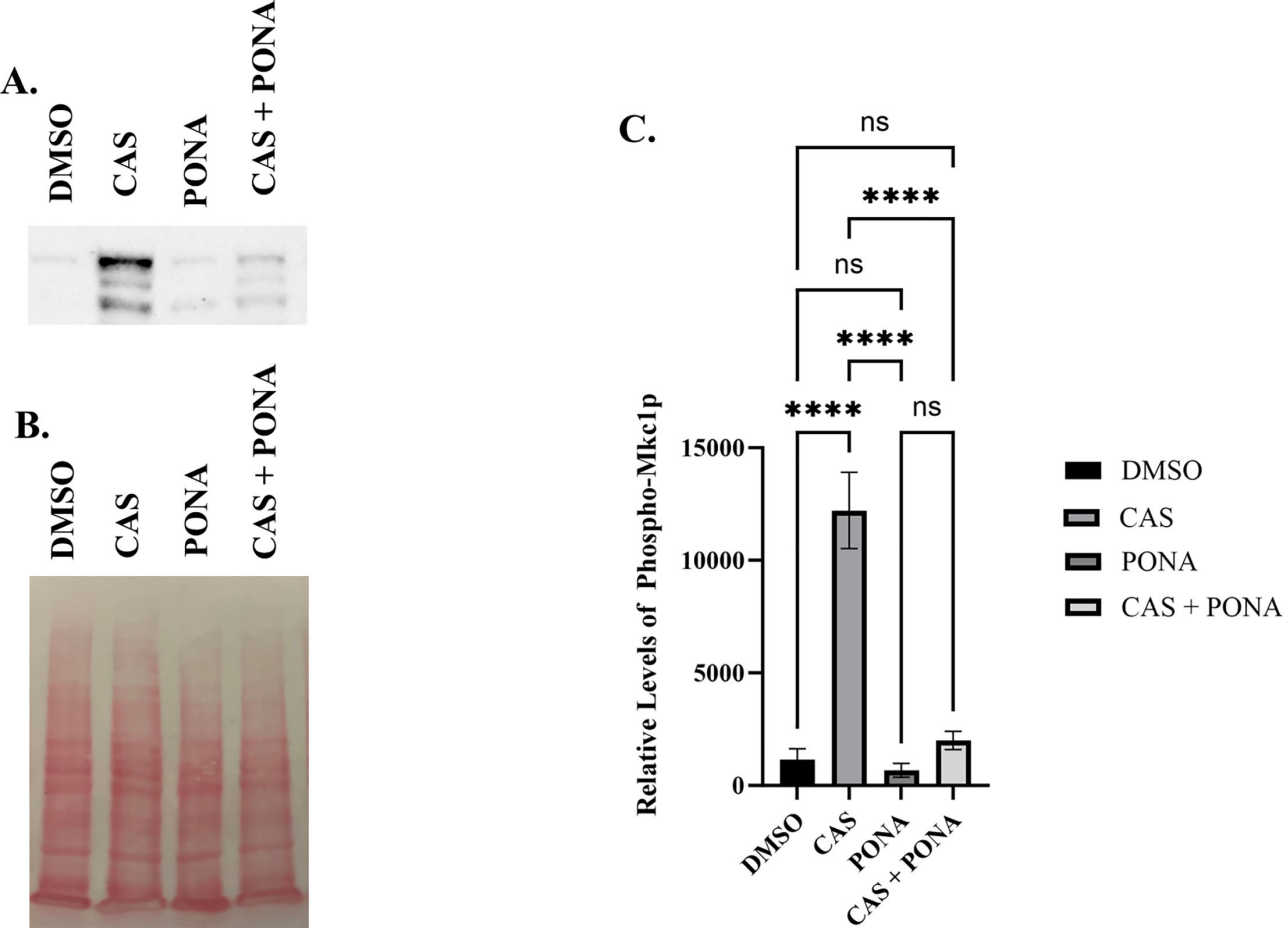
Ponatinib suppresses echinocandin-induced Mkc1p phosphorylation. (**A**) *C. albicans* SC5314 was grown for 2 hours in RPMI medium supplemented with 0.5% DMSO (vehicle), 0.05 μM caspofungin (CAS), 2.5 μM ponatinib (PONA), or both drugs in combination. Cell extracts were prepared, and levels of phospho-Mk1cp were quantified through immunoblot analysis with antiphospho-p44/42 and chemiluminescent substrate. (**B**) Ponceau S-stained membrane was used as a loading control. (**C**) Levels of phospho-Mk1cp detected were quantified by volumetric analysis. Data are the mean ± standard deviation of four independently performed experiments. Statistical significance was calculated using one-way ANOVA with Dunnett’s post-test. ****P < 0.0001.

**Fig 9:**
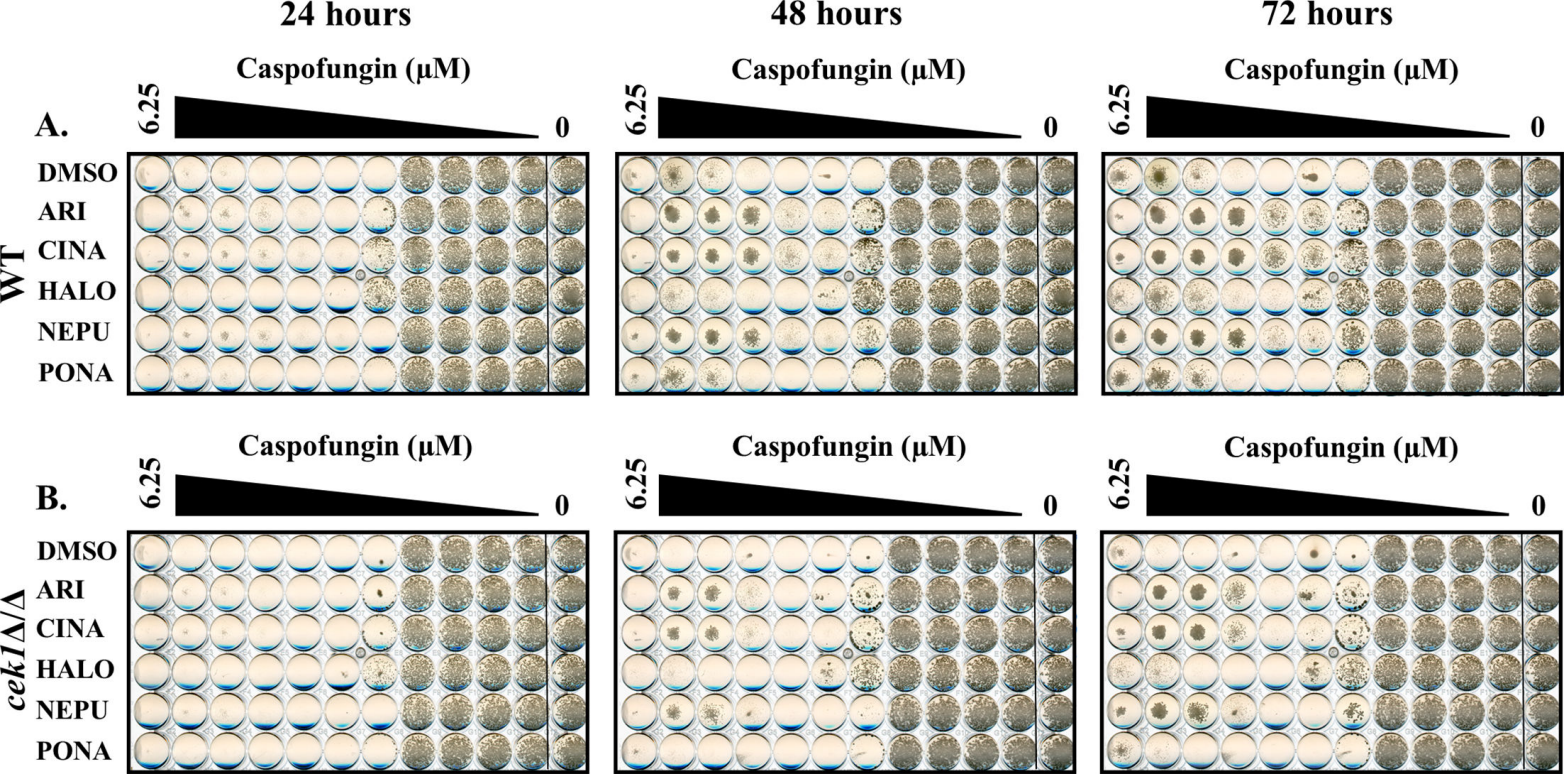
The Ceklp kinase is not required for echinocandin antagonism. The caspofungin susceptibility of wild-type (SN95) (**A**) and *cek1*Δ/Δ (**B**) *C. albicans* mutants was compared in RPMI-pH 7 (2% glucose) supplemented with either 5 μM aripiprazole (ARI), 5 μM cinacalcet (CINA), 5 μM haloperidol (HALO), 5 μM netupitant (NEPU), 2.5 μM ponatinib (PONA), or vehicle (DMSO). Plates were incubated at 35°C and imaged after 24, 48, and 72 hours. Images are representative of assays performed in biological duplicate.

Additional screens were performed with 4× MIC of caspofungin in the presence of each of the five antagonistic agents, non-responsive mutants identified (i.e., no antagonism) and follow-up dose responses conducted with caspofungin ± antagonist to confirm the phenotype. Between 8 and 13 mutants were confirmed to have reduced or no antagonistic responses for each antagonist (Fig. 6; Table 6). Substantial overlap was observed—with a total of 10 mutants identified as non-responsive to at least 4 of the five antagonists tested—suggesting shared underlying mechanisms. Most conspicuous were strains lacking *BCK1*, *MKK2*, and *MKC1* that encode components of the same MAP kinase signaling module, which has an established role in cell wall maintenance, caspofungin responses, and induction of hyphal growth through activation of the Cph1p transcription factor (29, 42). The importance of Cph1p was confirmed by the failure of a *cph1∆/∆* mutant to exhibit caspofungin antagonism with any of the five agents tested (Fig. 7). To determine if any of the antagonistic drugs affect activation of the Mkc1p pathway, extracts from SC5314 cells treated with each agent were probed with antiphospho-Mkc1p, but no major differences were apparent (data not shown). The presence of 0.25× MIC of caspofungin activated Mkc1p phosphorylation as expected (Fig. 8). Ponatinib substantially suppressed caspofungin-induced Mkc1p phosphorylation; however, the other four drugs had little effect. Thus, while the Mkc1p pathway is necessary for echinocandin antagonism with all five of the selected drugs, only one of the five agents had clear effects on its activity. The echinocandin antagonistic activity of four of the five drugs tested was also reduced in a *cek1Δ/Δ* mutant lacking an ERK-family protein kinase, which also has an established role in cell wall stress responses (43). However, the effect was less dramatic than observed for the Mkc1p-deficient mutant, being most obvious for ponatinib (Fig. 6 and 9). In addition, mutants lacking the *CST20* and *STE11* encoded kinases that function upstream of Cek1p were unaffected (Fig. S3), further indicating this pathway is less important.

**TABLE 6:** *Candida albicans* kinase mutants non-responsive to echinocandin antagonists

Mutant	Antagonists affected	Gene description^*a*^
*swe1*Δ/Δ	ARI, CINA, HALO, NEPU, PONA	Putative protein kinase with a role in the control of growth and morphogenesis
*ckb1*Δ/Δ	ARI, CINA, HALO, NEPU, PONA	Regulatory subunit of protein kinase CK2 (casein kinase II), beta subunit
*ckb2*Δ/Δ	ARI, CINA, HALO, NEPU, PONA	Regulatory subunit of protein kinase CK2 (casein kinase II), beta′ subunit
*mkc1*Δ/Δ	ARI, CINA, HALO, NEPU, PONA	MAP kinase; role in biofilm formation, contact-induced invasive filamentation, systemic virulence in mouse, cell wall structure/maintenance
*stt4*Δ/Δ	ARI, CINA, HALO, NEPU, PONA	Phosphatidylinositol-4-kinase, forms a complex with Ypp1p and Efr3p that is required for phosphatidylinositol-4-phosphate in the plasma membrane
*mkk2*Δ/Δ	ARI, CINA, NEPU, PONA	Ortholog of *S. cerevisiae* Mkk2p, MAP kinase kinase involved in signal transduction
*rad53*Δ/Δ	ARI, CINA, HALO, PONA	Protein involved in the regulation of DNA damage-induced filamentous growth, putative component of cell cycle checkpoint
*cek1*Δ/Δ	ARI, CINA, HALO, PONA	ERK-family protein kinase; required for wild-type yeast-hypha switch, mating efficiency, virulence in mice; Cst20p-Hst7p-Cek1p-Cph1p MAPK pathway
*bck1*Δ/Δ	ARI, CINA, HALO, NEPU	Ortholog of *S. cerevisiae* Bck1p, MAP kinase kinase kinase of the cell integrity pathway
*prk1*Δ/Δ	ARI, CINA, HALO, NEPU	Putative protein serine/threonine kinase
*kin2*Δ/Δ	ARI, CINA, HALO	Protein with similarity to *S. cerevisiae* Kin2p
*sak1*Δ/Δ	ARI, CINA	Serine/threonine protein kinase, acts as an upstream activating factor for the SNF1 complex that regulates responses to nutrient stress
*hsl1*Δ/Δ	ARI, CINA	Probable protein kinase involved in the determination of morphology during the cell cycle of both yeast-form and hyphal cells via regulation of Swe1p and Cdc28p

^*a*^ As described in *Candida* Genome Database.

### A core set of genes associated with xenobiotic response and hyphal growth is responsive to echinocandin antagonistic drugs

To provide additional insight into the mechanisms underlying the protective activity of the five selected antagonists, as well as their overall impact upon *C. albicans* physiology, we determined how each affects global patterns of gene transcription. SC5314 was grown at 35°C in RPMI medium supplemented with 5 µM of each antagonist (2.5 µM for ponatinib, which is growth inhibitory at 5 µM), or 0.5% DMSO (vehicle control); total RNA was extracted and subjected to high-throughput sequencing. Antagonist-responsive genes were defined as those in which the relative transcript read frequency was increased or decreased by ≥2-fold (*P* < 0.05) in the presence of drug versus vehicle control in each of two independent experiments. Between 590 and 42 gene transcripts were responsive to each individual drug with several altered by multiple drugs, indicating at least some similarities in their effects upon *C. albicans* physiology. We identified 17 transcripts as increased by at least four of the five antagonists, seven of which were associated with fluconazole resistance or regulation by the Tac1p transcription factor (Table 7; Table S1), including *CDR2*, which encodes an ATP-driven multi-drug efflux pump. An additional 10 of 43 transcripts elevated by any three of the five drugs were also associated with response or sensitivity to the azole antifungals or Tac1p-dependent regulation, including that of *CDR1*, encoding a second ATP-driven multi-drug efflux pump. Of the 22 transcripts repressed in the presence of at least four of the antagonists, nine are either required for *C. albicans* hyphal growth or exhibit hyphal-specific expression, including that of *ECE1*, which encodes candidalysin (44); *ALS3* and *HWP1*, both hyphal cell wall-associated adhesins that mediate attachment to mammalian cells (45, 46); and *UME6*, a transcription factor that promotes sustained hyphal growth (47). A further 11 of 41 transcripts downregulated by any three antagonists were also associated in some way with hyphal growth, including *CHT2* encoding a chitinase previously shown to be suppressed following echinocandin exposure (48). Many of these morphogenesis-related genes are known to be induced following activation of the Ras-cAMP-PkA-Efg1p-based signaling module, which plays a major role in the regulation of the yeast-hypha transition (49–51). Additionally, two transcripts previously identified as the most responsive to loss of cAMP production (52) were also affected, with *ASR1* induced by aripiprazole, cinacalcet, and netupitant and suppressed by ponatinib and *ASR2* induced by aripiprazole, cinacalcet, netupitant, and ponatinib. Curiously, the elevated transcription of the *ASR1* and *ASR2* genes is indicative of enhanced cAMP signaling, while suppression of the *ECE1*, *HWP1*, *ALS3*, and several other gene transcripts is consistent with diminished signaling through this pathway.

**TABLE 7:** *Candida albicans* gene transcripts responsive to four or more echinocandin antagonists tested^*a*^^*b*^

	Log_2_ of average fold change				
Gene	Aripiprazole	Cinacalcet	Haloperidol	Netupitant	Ponatinib
*RTA3*	**3.423**	**3.706**	**1.818**	**4.198**	**2.059**
*WH11*	**2.809**	**3.507**	**1.984**	**3.878**	**2.661**
*PGA26*	−2.107	−2.912	−3.453	−2.580	−2.667
*ALS3*	−2.545	−4.114	−4.510	−4.839	−1.146
*SOU2*	**3.336**	**3.436**	**3.420**	**2.476**	NA
*orf19.6502*	**2.249**	**2.483**	**1.961**	**2.382**	NA
*RSN1*	**1.684**	**1.663**	**2.670**	**1.465**	NA
*GCY1*	**1.436**	**1.419**	**1.719**	**1.389**	NA
*LTV1*	−1.660	−1.542	−1.349	−1.237	NA
*orf19.5710*	−1.232	−1.617	−1.369	−1.737	NA
*SEO1*	−1.889	−2.155	−1.872	−2.312	NA
*orf19.3475*	−1.724	−1.847	−3.245	−1.588	NA
*DEF1*	−2.213	−2.071	−1.843	−2.361	NA
*UME6*	−2.695	−3.967	−2.154	−4.213	NA
*IHD1*	−3.511	−5.346	−2.929	−4.296	NA
*ECE1*	−3.211	−5.788	−2.523	−5.915	NA
*HWP1*	−3.118	−5.969	−2.933	−5.876	NA
*CFL11*	−4.302	−4.847	−4.012	−4.788	NA
*CDR2*	**5.776**	**6.432**	NA	**6.186**	**4.105**
*orf19.4886*	**2.527**	**3.187**	NA	**2.959**	**2.313**
*OYE23*	**1.828**	**2.208**	NA	**2.751**	**2.596**
*ASR2*	**2.287**	**2.772**	NA	**3.218**	**1.074**
*orf19.4216*	**2.077**	**2.556**	NA	**2.254**	**1.857**
*orf19.86*	**2.034**	**2.093**	NA	**2.503**	**1.654**
*HSP12*	**1.881**	**2.355**	NA	**2.131**	**1.565**
*orf19.4907*	**1.927**	**1.987**	NA	**2.438**	**1.227**
*IFE1*	**1.498**	**1.855**	NA	**2.240**	**1.880**
*SOD5*	−1.790	−2.456	NA	−2.170	−2.946
*orf19.6200*	−3.348	−3.614	NA	−4.799	−3.497
*orf19.3406*	NA	−1.193	−1.382	−1.206	−1.811
*PHO89*	NA	−2.685	−2.514	−2.941	−1.850
*GIT1*	NA	−2.433	−3.220	−2.046	−2.393

^*a*^ NA, non applicable (i.e., no significant change in transcript abundance compared to vehicle-treated cells).

^*b*^ Upregulated gene transcripts are shown in bold typeface.

### Echinocandin antagonists suppress *C. albicans* hyphal growth

**Fig 10:**
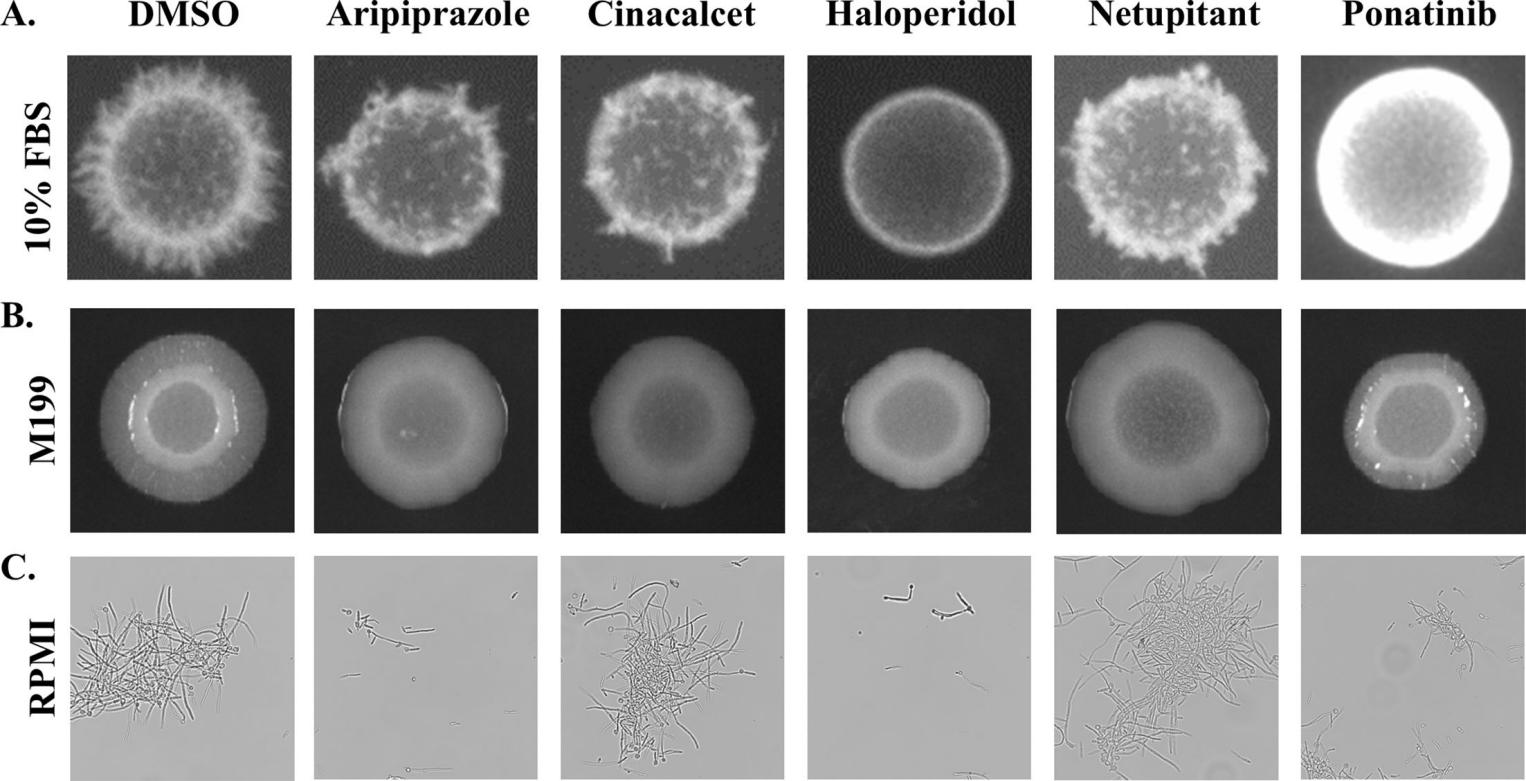
Several echinocandin antagonists suppress *Candida albicans* hyphal growth. (**A and B**) SC5314 was suspended at 2 × 10^6^ cells/mL, and 2 μL was spotted to 10% fetal bovine serum (**A**) or M199 (**B**) agar supplemented with either 5 μM aripiprazole, 5 μM haloperidol, 5 μM netupitant, 5 μM cinacalcet, 2.5 μM ponatinib, or vehicle (DMSO). Colonies were then imaged after 96 hours at 37°C. (**C**) SC5314 was suspended in liquid RPMI medium supplemented with the same drug concentrations as in **A and B**. After 6 hours of incubation, cells were fixed in 10% formalin, and cell morphology was observed microscopically. Images are representative of assays performed in biological triplicate.

**Fig 11:**
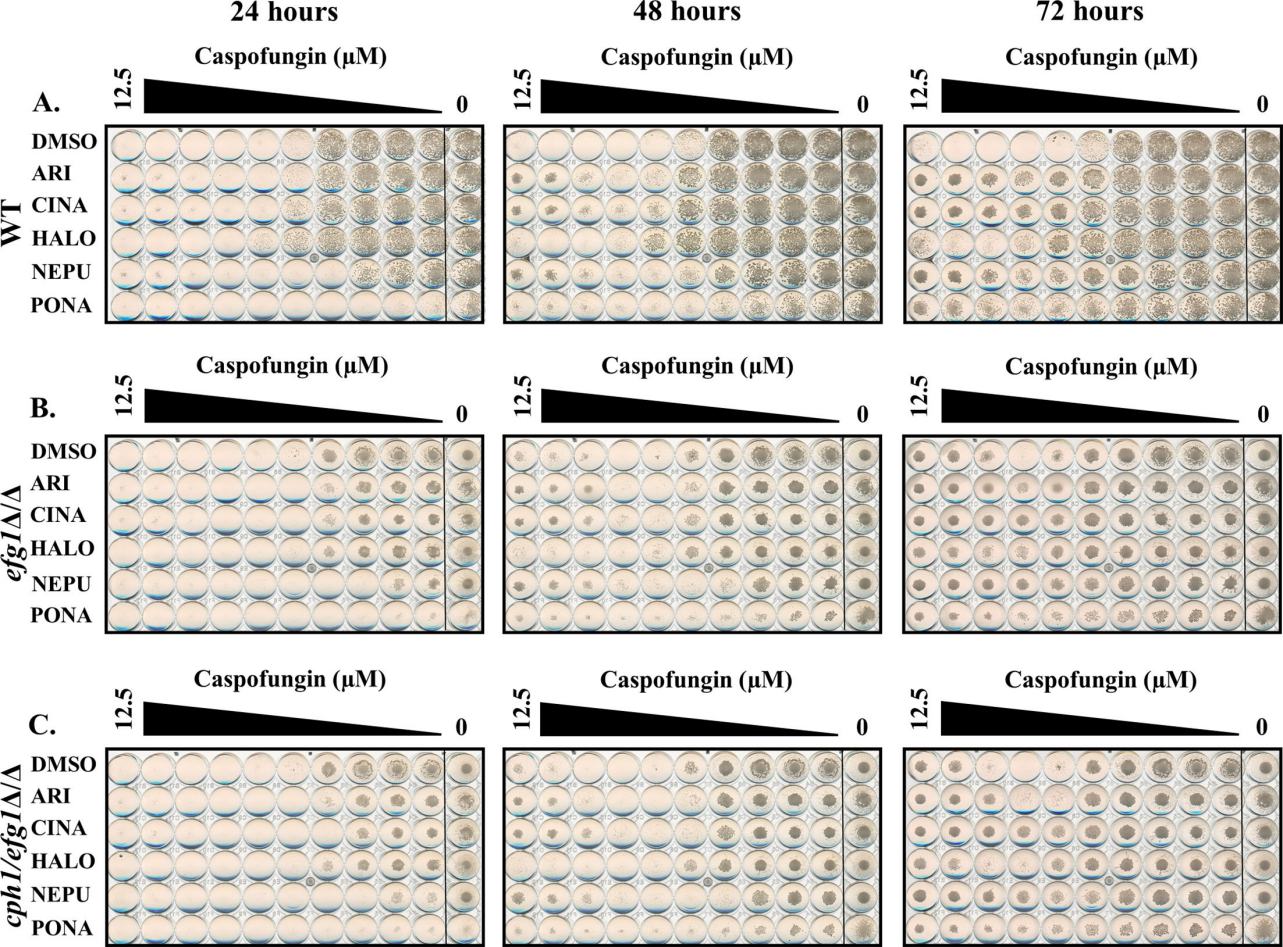
Deletion of *EFG1* induces echinocandin tolerance in *Candida albicans*. The caspofungin sensitivity of wild-type (SC5314, (**A**), *efg1*Δ/Δ (**B**), and *efg1*Δ/Δ*cph1*Δ/Δ (**C**) *C. albicans* strains was compared in RPMI-pH 7 (2% glucose) supplemented with either 5 μM aripiprazole (ARI), 5 μM cinacalcet (CINA), 5 μM haloperidol (HALO), 5 μM netupitant (NEPU), 2.5 μM ponatinib (PONA), or vehicle (DMSO). Plates were incubated at 35°C and imaged after 24, 48, and 72 hours. Images are representative of assays performed in biological duplicate.

Given the impact of the echinocandin antagonists upon morphogenesis-responsive transcripts, the influence of each upon *C. albicans* hyphal growth was examined. All five echinocandin antagonists suppressed *C. albicans* hyphal growth on either 10% fetal bovine serum or M199 agar to varying extents (Fig. 10A and B), with haloperidol and ponatinib having the greatest effect. In liquid RPMI medium at 35°C, aripiprazole moderately suppressed hyphal formation, as we have previously reported (34), reducing the proportion of cells forming hyphae as well as the length of those that form. Haloperidol and ponatinib also significantly reduced hyphal length under these conditions (Fig. 10C), while netupitant and cinacalcet had no obvious effect. To further probe the connection between the cAMP-PkA-Efg1p morphogenesis-related signaling pathway and echinocandin antagonism, an *efg1∆/∆* mutant was utilized. Dose-response experiments revealed that the *efg1∆/∆* mutant had elevated levels of paradoxical growth compared to wild-type that was unaffected by any of the aforementioned five antagonists (Fig. 11). The mutant also exhibited substantial levels of tolerance to both anidulafungin and micafungin (data not shown). These data indicate that the morphogenesis-related transcription factor Efg1p confers susceptibility to caspofungin. Furthermore, the data are consistent with the five echinocandin antagonists acting through suppression of Efg1p activity.

## DISCUSSION

The studies described herein have revealed a substantial number of drug-fungus interactions that directly or indirectly affect *C. albicans* susceptibility to the ordinarily cidal echinocandin antifungals. Several points are notable: the MIC determined at the 24 hour time point was not (in most cases) significantly altered by the antagonistic drug, but rather residual growth observed above the MIC at 48 and 72 hours was dramatically enhanced. Based on this phenotype, as well as viability counts following caspofungin exposure, we have classified the phenomenon as a form of tolerance that promotes survival of the fungal cells, rather than resistance. For four of the five antagonists examined in detail, this also appeared consistent with an enhanced form of the paradoxical effect previously described at high concentrations of the echinocandins. However, paradoxical growth is principally observed with caspofungin, less commonly with micafungin or anidulafungin (35, 53), and is more apparent in some *C. albicans* isolates over others. In contrast, the antagonistic activities we have described affect *C. albicans* response to all three echinocandins, and their activity does not seem restricted to specific isolates. Thus, it remains uncertain how the drug-induced echinocandin antagonism observed within this study is qualitatively related to the previously described paradoxical phenotype.

Our findings suggest that the antagonism observed depends upon a complex response requiring multiple signaling pathways that have been fairly well characterized with respect to their roles in regulating yeast-hypha morphogenesis and *C. albicans* response to cell wall stress (43, 54, 55). Specifically, the Mkc1p-mitogen-activated protein kinase (MAPK) signaling module, which acts upon the Cph1p transcription factor (56), is crucial for the echinocandin tolerance induced by the antagonists. *CKB1* and *CKB2*, both encoding regulatory subunits of casein kinase 2, were also identified as non-responsive to the caspofungin antagonists. Signaling through the calcium-dependent calcineurin (CN) phosphatase pathway is known to be important for fungal survival upon exposure to both azole and echinocandin antifungals (57). Blocking CN signaling with either cyclosporin A or FK506 or using a *C. albicans cnb1∆/∆* mutant, lacking the B regulatory subunit of CN, completely eliminates the antagonistic activity of all five drugs (Fig. S4 and S5). In contrast, four of the five antagonists examined (except haloperidol) promote caspofungin tolerance in a *crz1∆/∆* mutant (Fig. S5), lacking one of the major CN-responsive transcription factors (58). Thus, Crz1p-independent CN signaling is also required for echinocandin antagonism. Unexpectedly, we observed that a *C. albicans* mutant lacking the Hog1p MAP kinase, also with established roles in cell wall stress responses, is highly caspofungin tolerant. However, aripiprazole, cinacalcet, netupitant, and ponatinib all further elevate the growth of this mutant in the presence of supra-MIC concentrations of caspofungin (Fig. S6), suggesting they act independently of Hog1p. Together, these results indicate that the echinocandin antagonists tested act through a complex mechanism that requires both Mkc1p, CK2, and Crz1p-independent functions of CN. The similarities in the pathway requirements of the five antagonists studied in detail suggest a largely shared molecular mechanism, which is surprising, given the diversity of their chemical structure and of their targets within the mammalian host. No clear *C. albicans* homologs of the known targets of aripiprazole, cinacalcet, haloperidol, or netupitant could be identified (Table 1), while the *ABL1*-encoded target of ponatinib shares only general homology to a large number of kinases encoded by the *C. albicans* genome. Thus, the capacity to sense these unrelated small molecules and produce a seemingly common functional response with respect to the tolerance phenotype is cryptic and suggests the presence of xenobiotic sensing modules and/or receptors capable of promiscuous interactions with small molecules. Indeed, analysis of transcriptional responses indicated that a significant number of echinocandin antagonists affect the transcription of Tac1p-responsive genes. The transcription factor Tac1p and close relatives from other species can directly bind several medically important molecules, most notably fluphenazine (59), to activate transcription of genes encoding the Cdr1p and Cdr2p efflux pumps. We also recently reported that a substantial proportion of drugs identified as fluconazole antagonists require Tac1p for activity (28), suggesting that this response module is able to sense a much broader array of molecular species than previously appreciated. However, in contrast to the fluconazole antagonists, most of which required Tac1p for activity, Tac1p is not required for the echinocandin antagonism described herein (Fig. S7), and its activation is therefore likely incidental. Given that the echinocandins are believed to engage β(1,3)-D-glucan synthase at the cell surface, it is unsurprising that Tac1p-mediated activation of *CDR1* and *CDR2* transcription, or of the *MDR1*-encoded efflux pump (Fig. S8), does not account for the observed antagonism. As such, Tac1p activation seems to define a more general response to encountering xenobiotics. Nonetheless, it is clear that *C. albicans* possesses systems that function to detect and respond to an impressive variety of molecular entities.

The suppression of hyphal growth and hyphal-associated gene transcription by the five antagonists studied raises questions of how cellular morphotype may affect *C. albicans* echinocandin sensitivity. Future work should explore if modulation of yeast-hypha morphogenesis can provide a strategy for *C. albicans* to mitigate the consequences of echinocandin exposure and determine if yeast and hyphal forms are similarly susceptible to echinocandin-induced cell death.

A major rationale for conducting the antagonism screen was to identify factors that can help explain the often-unpredictable response of individual patients to echinocandin therapy; i.e., could patients treated with one or more antagonistic medications be less responsive to antifungal treatment and/or more prone to recurrence? In a broader context, this study raises questions of how biologically active molecules, including the medications consumed by colonized or infected individuals, affect fungal physiology and thus potentially the equilibrium of the host-pathogen interaction. Drug-fungus interactions, for example, could alter the outcome of infection by: (i) causing profound physiological dysfunction that compromises fungal viability, fitness, or pathogenicity; (ii) changing the surface characteristics of the fungus in ways that alter its immunogenicity, binding/activation of complement, or sensitivity to host-derived antimicrobial peptides; or (iii) altering fungal sensitivity to antifungal drugs, as is the focus of this study. However, the highly variable, often transient, and combinatorial nature of medications usually provided to patients at risk of invasive fungal infections, specific clinical circumstances (including the nature and severity of immune dysfunction), site of infection, causative species, and potentially genetic factors of both the host and pathogen are likely to make it difficult, if not impossible, to discern the impact of any individual drug upon antifungal efficacy based on clinical observations alone. These factors are also likely to provide significant constraints on the power of clinical studies to reliably detect the impact of individual medications upon therapeutic efficacy, or the outcome of infection, especially given the relatively small numbers of infected patients that may be treated with a given antagonistic medication. Nonetheless, these limitations do not mean that such drug-fungus interactions have no impact, and it is possible they may help explain the often-poor correlation between the results of *in vitro* antifungal susceptibility tests and clinical efficacy. A more reliable strategy may be to use animal models of infection that enable control of many of the confounding variables.

Several key factors are likely to determine if the antagonistic agents identified within this study affect echinocandin efficacy within mammalian tissue. First, it is important to consider the effects of each drug at therapeutically relevant drug concentrations, which—in the context of disseminated infection—most investigators will take to be unbound blood serum concentrations from patients on a standard dosing regimen. Based on this parameter, only aripiprazole alters *C. albicans* echinocandin sensitivity within or at least close to clinically relevant concentration ranges (Table S2) (60–62). However, it is the available concentration of the antifungal as well as the antagonistic agent at the site of infection that is most relevant, and tissue concentrations can differ markedly from unbound serum concentrations. Additionally, acute inflammation, tissue dissolution, and protein digestion by fungal enzymes within foci of infection could have further effects, and drug concentrations at the site of fungal infection are unknown. Finally, *Candida* spp. naturally colonize the GI tract, where the concentration of orally administered drugs (or those excreted via the GI tract) is potentially much higher. Either way, the antagonists identified through this study can provide valuable chemical probes to better understand the molecular mechanisms that affect fungal survival following echinocandin exposure.

## SUPPLEMENTAL MATERIAL

Supplemental figures aac.00484-25-s0001.pdf Fig. S1 to S4.

Supplemental material aac.00484-25-s0002.pdf Fig. S5 to S8.

Supplemental tables aac.00484-25-s0003.pdf Tables S1 to S3.

ASM does not own the copyrights to Supplemental Material that may be linked to, or accessed through, an article. The authors have granted ASM a non-exclusive, world-wide license to publish the Supplemental Material files. Please contact the corresponding author directly for reuse.

## References

[1] Benedict K, Jackson BR, Chiller T, Beer KD. 2019. Estimation of direct healthcare costs of fungal diseases in the United States. Clin Infect Dis 68:1791–1797. doi:10.1093/cid/ciy776 30204844 PMC6409199

[2] Horn DL, Neofytos D, Anaissie EJ, Fishman JA, Steinbach WJ, Olyaei AJ, Marr KA, Pfaller MA, Chang C-H, Webster KM. 2009. Epidemiology and outcomes of candidemia in 2019 patients: data from the prospective antifungal therapy alliance registry. Clin Infect Dis 48:1695–1703. doi:10.1086/599039 19441981

[3] Jarvis WR. 1995. Epidemiology of nosocomial fungal infections, with emphasis on Candida species. Clin Infect Dis 20:1526–1530. doi:10.1093/clinids/20.6.1526 7548503

[4] Pittet D, Wenzel RP. 1995. Nosocomial bloodstream infections. Secular trends in rates, mortality, and contribution to total hospital deaths. Arch Intern Med 155:1177–1184. doi:10.1001/archinte.155.11.1177 7763123

[5] Brown GD, Denning DW, Gow NAR, Levitz SM, Netea MG, White TC. 2012. Hidden killers: human fungal infections. Sci Transl Med 4:165rv13. doi:10.1126/scitranslmed.3004404 23253612

[6] Edmond MB, Wallace SE, McClish DK, Pfaller MA, Jones RN, Wenzel RP. 1999. Nosocomial bloodstream infections in United States hospitals: a three‐year analysis. Clin Infect Dis 29:239–244. doi:10.1086/520192 10476719

[7] Sobel JD. 2007. Vulvovaginal candidosis. Lancet 369:1961–1971. doi:10.1016/S0140-6736(07)60917-9 17560449

[8] Chen SC, Slavin MA, Sorrell TC. 2011. Echinocandin antifungal drugs in fungal infections: a comparison. Drugs 71:11–41. doi:10.2165/11585270-000000000-00000 21175238

[9] Betts RF, Nucci M, Talwar D, Gareca M, Queiroz-Telles F, Bedimo RJ, Herbrecht R, Ruiz-Palacios G, Young J-AH, Baddley JW, Strohmaier KM, Tucker KA, Taylor AF, Kartsonis NA, Caspofungin High-Dose Study Group. 2009. A multicenter, double-blind trial of a high-dose caspofungin treatment regimen versus a standard caspofungin treatment regimen for adult patients with invasive candidiasis. Clin Infect Dis 48:1676–1684. doi:10.1086/598933 19419331

[10] Mora-Duarte J, Betts R, Rotstein C, Colombo AL, Thompson-Moya L, Smietana J, Lupinacci R, Sable C, Kartsonis N, Perfect J, Caspofungin Invasive Candidiasis Study Group. 2002. Comparison of caspofungin and amphotericin B for invasive candidiasis. N Engl J Med 347:2020–2029. doi:10.1056/NEJMoa021585 12490683

[11] Pappas PG, Rotstein CMF, Betts RF, Nucci M, Talwar D, De Waele JJ, Vazquez JA, Dupont BF, Horn DL, Ostrosky-Zeichner L, Reboli AC, Suh B, Digumarti R, Wu C, Kovanda LL, Arnold LJ, Buell DN. 2007. Micafungin versus caspofungin for treatment of candidemia and other forms of invasive candidiasis. Clin Infect Dis 45:883–893. doi:10.1086/520980 17806055

[12] Kullberg BJ, Viscoli C, Pappas PG, Vazquez J, Ostrosky-Zeichner L, Rotstein C, Sobel JD, Herbrecht R, Rahav G, Jaruratanasirikul S, Chetchotisakd P, Van Wijngaerden E, De Waele J, Lademacher C, Engelhardt M, Kovanda L, Croos-Dabrera R, Fredericks C, Thompson GR. 2019. Isavuconazole versus caspofungin in the treatment of candidemia and other invasive candida infections: the ACTIVE trial. Clin Infect Dis 68:1981–1989. doi:10.1093/cid/ciy827 30289478

[13] Walsh TJ, Teppler H, Donowitz GR, Maertens JA, Baden LR, Dmoszynska A, Cornely OA, Bourque MR, Lupinacci RJ, Sable CA, dePauw BE. 2004. Caspofungin versus liposomal amphotericin B for empirical antifungal therapy in patients with persistent fever and neutropenia. N Engl J Med 351:1391–1402. doi:10.1056/NEJMoa040446 15459300

[14] Pfaller MA. 2012. Antifungal drug resistance: mechanisms, epidemiology, and consequences for treatment. Am J Med 125:S3–13. doi:10.1016/j.amjmed.2011.11.001 22196207

[15] Sanglard D, Odds FC. 2002. Resistance of Candida species to antifungal agents: molecular mechanisms and clinical consequences. Lancet Infect Dis 2:73–85. doi:10.1016/s1473-3099(02)00181-0 11901654

[16] Rex JH. 2008*.* Reference method for broth dilution antifungal susceptibility testing of filamentous fungi: approved standard. Clinical and Laboratory Standards Institute.

[17] Baddley JW, Patel M, Bhavnani SM, Moser SA, Andes DR. 2008. Association of fluconazole pharmacodynamics with mortality in patients with candidemia. Antimicrob Agents Chemother 52:3022–3028. doi:10.1128/AAC.00116-08 18591269 PMC2533442

[18] Pfaller MA, Boyken L, Hollis RJ, Kroeger J, Messer SA, Tendolkar S, Diekema DJ. 2008. In vitro susceptibility of invasive isolates of Candida spp. to anidulafungin, caspofungin, and micafungin: six years of global surveillance. J Clin Microbiol 46:150–156. doi:10.1128/JCM.01901-07 18032613 PMC2224271

[19] Pfaller MA, Boyken L, Hollis RJ, Messer SA, Tendolkar S, Diekema DJ. 2006. In vitro susceptibilities of Candida spp. to caspofungin: four years of global surveillance. J Clin Microbiol 44:760–763. doi:10.1128/JCM.44.3.760-763.2006 16517851 PMC1393154

[20] Garey KW, Rege M, Pai MP, Mingo DE, Suda KJ, Turpin RS, Bearden DT. 2006. Time to initiation of fluconazole therapy impacts mortality in patients with candidemia: a multi-institutional study. Clin Infect Dis 43:25–31. doi:10.1086/504810 16758414

[21] MacCallum DM, Odds FC. 2004. Need for early antifungal treatment confirmed in experimental disseminated Candida albicans infection. Antimicrob Agents Chemother 48:4911–4914. doi:10.1128/AAC.48.12.4911-4914.2004 15561880 PMC529222

[22] Van t Wout JW, Mattie H, van Furth R. 1989. Comparison of the efficacies of amphotericin B, fluconazole, and itraconazole against a systemic Candida albicans infection in normal and neutropenic mice. Antimicrob Agents Chemother 33:147–151. doi:10.1128/AAC.33.2.147 2541654 PMC171446

[23] Robbins N, Spitzer M, Yu T, Cerone RP, Averette AK, Bahn Y-S, Heitman J, Sheppard DC, Tyers M, Wright GD. 2015. An antifungal combination matrix identifies a rich pool of adjuvant molecules that enhance drug activity against diverse fungal pathogens. Cell Rep 13:1481–1492. doi:10.1016/j.celrep.2015.10.018 26549450 PMC4654976

[24] Cui J, Ren B, Tong Y, Dai H, Zhang L. 2015. Synergistic combinations of antifungals and anti-virulence agents to fight against Candida albicans. Virulence 6:362–371. doi:10.1080/21505594.2015.1039885 26048362 PMC4601232

[25] Spitzer M, Griffiths E, Blakely KM, Wildenhain J, Ejim L, Rossi L, De Pascale G, Curak J, Brown E, Tyers M, Wright GD. 2011. Cross-species discovery of syncretic drug combinations that potentiate the antifungal fluconazole. Mol Syst Biol 7:499. doi:10.1038/msb.2011.31 21694716 PMC3159983

[26] Zhang L, Yan K, Zhang Y, Huang R, Bian J, Zheng C, Sun H, Chen Z, Sun N, An R, et al.. 2007. High-throughput synergy screening identifies microbial metabolites as combination agents for the treatment of fungal infections. Proc Natl Acad Sci USA 104:4606–4611. doi:10.1073/pnas.0609370104 17360571 PMC1838648

[27] Butts A, Reitler P, Ge W, Fortwendel JR, Palmer GE. 2018. Commonly used oncology drugs decrease antifungal effectiveness against Candida and Aspergillus species. Antimicrob Agents Chemother 62:e00504-18. doi:10.1128/AAC.00504-18 29712657 PMC6021626

[28] Butts A, Reitler P, Nishimoto AT, DeJarnette C, Estredge LR, Peters TL, Veve MP, Rogers PD, Palmer GE. 2019. A systematic screen reveals a diverse collection of medications that induce antifungal resistance in Candida species. Antimicrob Agents Chemother 63:e00054-19. doi:10.1128/AAC.00054-19 30858206 PMC6496105

[29] Kramara J, Kim M-J, Ollinger TL, Ristow LC, Wakade RS, Zarnowski R, Wellington M, Andes DR, Mitchell AG, Krysan DJ. 2024. Systematic analysis of the Candida albicans kinome reveals environmentally contingent protein kinase-mediated regulation of filamentation and biofilm formation in vitro and in vivo mBio 15:e0124924. doi:10.1128/mbio.01249-24 38949302 PMC11323567

[30] Andrade C, Scores Z. 2021. Z scores, standard scores, and composite test scores explained. Indian J Psychol Med 43:555–557. doi:10.1177/02537176211046525 35210687 PMC8826187

[31] Luna-Tapia A, Tournu H, Peters TL, Palmer GE. 2016. Endosomal trafficking defects can induce calcium-dependent azole tolerance in Candida albicans. Antimicrob Agents Chemother 60:7170–7177. doi:10.1128/AAC.01034-16 27645241 PMC5118996

[32] Butts A, DeJarnette C, Peters TL, Parker JE, Kerns ME, Eberle KE, Kelly SL, Palmer GE. 2017. Target abundance-based fitness screening (TAFiS) facilitates rapid identification of target-specific and physiologically active chemical probes. mSphere 2:e00379-17. doi:10.1128/mSphere.00379-17 PMC5628291 28989971

[33] Johnson DL, Kumar R, Kakhniashvili D, Pfeffer LM, Laribee RN. 2023. Ccr4-not ubiquitin ligase signaling regulates ribosomal protein homeostasis and inhibits 40S ribosomal autophagy. bioRxiv. doi:10.1101/2023.08.28.555095 PMC11357857 39025453

[34] Reitler P, Regan J, DeJarnette C, Srivastava A, Carnahan J, Tucker KM, Meibohm B, Peters BM, Palmer GE. 2024. The atypical antipsychotic aripiprazole alters the outcome of disseminated Candida albicans infections. Infect Immun 92:e0007224. doi:10.1128/iai.00072-24 38899880 PMC11238555

[35] Stevens DA, Espiritu M, Parmar R. 2004. Paradoxical effect of caspofungin: reduced activity against Candida albicans at high drug concentrations. Antimicrob Agents Chemother 48:3407–3411. doi:10.1128/AAC.48.9.3407-3411.2004 15328104 PMC514730

[36] Meletiadis J, Pournaras S, Roilides E, Walsh TJ. 2010. Defining fractional inhibitory concentration index cutoffs for additive interactions based on self-drug additive combinations, Monte Carlo simulation analysis, and in vitro-in vivo correlation data for antifungal drug combinations against Aspergillus fumigatus. Antimicrob Agents Chemother 54:602–609. doi:10.1128/AAC.00999-09 19995928 PMC2812160

[37] Berenbaum MC. 1978. A method for testing for synergy with any number of agents. J Infect Dis 137:122–130. doi:10.1093/infdis/137.2.122 627734

[38] Bliss CI. 1939. The toxicity of poisons applied jointly. Ann Appl Biol 26:585–615. doi:10.1111/j.1744-7348.1939.tb06990.x

[39] White TC, Pfaller MA, Rinaldi MG, Smith J, Redding SW. 1997. Stable azole drug resistance associated with a substrain of Candida albicans from an HIV-infected patient. Oral Dis 3 Suppl 1:S102–9. doi:10.1111/j.1601-0825.1997.tb00336.x 9456667

[40] England CG, Ehlerding EB, Cai W. 2016. NanoLuc: a small luciferase is brightening up the field of bioluminescence. Bioconjug Chem 27:1175–1187. doi:10.1021/acs.bioconjchem.6b00112 27045664 PMC4871753

[41] Cheetham J, Smith DA, da Silva Dantas A, Doris KS, Patterson MJ, Bruce CR, Quinn J. 2007. A single MAPKKK regulates the Hog1 MAPK pathway in the pathogenic fungus Candida albicans. Mol Biol Cell 18:4603–4614. doi:10.1091/mbc.e07-06-0581 17804815 PMC2043575

[42] Kumamoto CA. 2005. A contact-activated kinase signals Candida albicans invasive growth and biofilm development. Proc Natl Acad Sci USA 102:5576–5581. doi:10.1073/pnas.0407097102 15800048 PMC556227

[43] Ene IV, Walker LA, Schiavone M, Lee KK, Martin-Yken H, Dague E, Gow NAR, Munro CA, Brown AJP. 2015. Cell wall remodeling enzymes modulate fungal cell wall elasticity and osmotic stress resistance. mBio 6:e00986. doi:10.1128/mBio.00986-15 26220968 PMC4551979

[44] Moyes DL, Wilson D, Richardson JP, Mogavero S, Tang SX, Wernecke J, Höfs S, Gratacap RL, Robbins J, Runglall M, et al.. 2016. Candidalysin is a fungal peptide toxin critical for mucosal infection. Nature 532:64–68. doi:10.1038/nature17625 27027296 PMC4851236

[45] Liu Y, Filler SG. 2011. Candida albicans Als3, a multifunctional adhesin and invasin. Eukaryot Cell 10:168–173. doi:10.1128/EC.00279-10 21115738 PMC3067396

[46] Nobile CJ, Nett JE, Andes DR, Mitchell AP. 2006. Function of Candida albicans adhesin Hwp1 in biofilm formation. Eukaryot Cell 5:1604–1610. doi:10.1128/EC.00194-06 17030992 PMC1595337

[47] Banerjee M, Thompson DS, Lazzell A, Carlisle PL, Pierce C, Monteagudo C, López-Ribot JL, Kadosh D. 2008. UME6, a novel filament-specific regulator of Candida albicans hyphal extension and virulence. Mol Biol Cell 19:1354–1365. doi:10.1091/mbc.e07-11-1110 18216277 PMC2291399

[48] Yadav A, Sah SK, Perlin DS, Rustchenko E. 2024. Candida albicans genes modulating echinocandin susceptibility of caspofungin-adapted mutants are constitutively expressed in clinical isolates with intermediate or full resistance to echinocandins. J Fungi 10:224. doi:10.3390/jof10030224 PMC10971431 38535232

[49] Huang G, Huang Q, Wei Y, Wang Y, Du H. 2019. Multiple roles and diverse regulation of the Ras/cAMP/protein kinase A pathway in Candida albicans. Mol Microbiol 111:6–16. doi:10.1111/mmi.14148 30299574

[50] Glazier VE. 2022. EFG1, everyone’s favorite gene in Candida albicans: a comprehensive literature review. Front Cell Infect Microbiol 12:855229. doi:10.3389/fcimb.2022.855229 35392604 PMC8980467

[51] Kornitzer D. 2019. Regulation of Candida albicans hyphal morphogenesis by endogenous signals. J Fungi 5:21. doi:10.3390/jof5010021 PMC6463138 30823468

[52] Harcus D, Nantel A, Marcil A, Rigby T, Whiteway M. 2004. Transcription profiling of cyclic AMP signaling in Candida albicans. Mol Biol Cell 15:4490–4499. doi:10.1091/mbc.e04-02-0144 15269278 PMC519143

[53] Chamilos G, Lewis RE, Albert N, Kontoyiannis DP. 2007. Paradoxical effect of echinocandins across Candida species in vitro: evidence for echinocandin-specific and Candida species-related differences. Antimicrob Agents Chemother 51:2257–2259. doi:10.1128/AAC.00095-07 17438060 PMC1891358

[54] Gow NAR, van de Veerdonk FL, Brown AJP, Netea MG. 2011. Candida albicans morphogenesis and host defence: discriminating invasion from colonization. Nat Rev Microbiol 10:112–122. doi:10.1038/nrmicro2711 22158429 PMC3624162

[55] Ibe C, Munro CA. 2021. Fungal cell wall proteins and signaling pathways form a cytoprotective network to combat stresses. J Fungi 7:739. doi:10.3390/jof7090739 PMC8466366 34575777

[56] Heilmann CJ, Sorgo AG, Mohammadi S, Sosinska GJ, de Koster CG, Brul S, de Koning LJ, Klis FM. 2013. Surface stress induces a conserved cell wall stress response in the pathogenic fungus Candida albicans. Eukaryot Cell 12:254–264. doi:10.1128/EC.00278-12 23243062 PMC3571293

[57] Juvvadi PR, Lee SC, Heitman J, Steinbach WJ. 2017. Calcineurin in fungal virulence and drug resistance: prospects for harnessing targeted inhibition of calcineurin for an antifungal therapeutic approach. Virulence 8:186–197. doi:10.1080/21505594.2016.1201250 27325145 PMC5354160

[58] Karababa M, Valentino E, Pardini G, Coste AT, Bille J, Sanglard D. 2006. CRZ1, a target of the calcineurin pathway in Candida albicans. Mol Microbiol 59:1429–1451. doi:10.1111/j.1365-2958.2005.05037.x 16468987

[59] Liu Z, Myers LC. 2017. Mediator tail module is required for Tac1-activated CDR1 expression and azole resistance in Candida albicans. Antimicrob Agents Chemother 61:11. doi:10.1128/AAC.01342-17 PMC5655045 28807920

[60] Andes D, van Ogtrop M. 1999. Characterization and quantitation of the pharmacodynamics of fluconazole in a neutropenic murine disseminated candidiasis infection model. Antimicrob Agents Chemother 43:2116–2120. doi:10.1128/AAC.43.9.2116 10471550 PMC89432

[61] Uematsu T, Matsuno H, Sato H, Hirayama H, Hasegawa K, Nakashima M. 1992. Steady-state pharmacokinetics of haloperidol and reduced haloperidol in schizophrenic patients: analysis of factors determining their concentrations in hair. J Pharm Sci 81:1008–1011. doi:10.1002/jps.2600811010 1432610

[62] Nicolini FE, Basak GW, Kim D-W, Olavarria E, Pinilla-Ibarz J, Apperley JF, Hughes T, Niederwieser D, Mauro MJ, Chuah C, Hochhaus A, Martinelli G, DerSarkissian M, Duh MS, McGarry LJ, Kantarjian HM, Cortes JE. 2017. Overall survival with ponatinib versus allogeneic stem cell transplantation in Philadelphia chromosome-positive leukemias with the T315I mutation. Cancer 123:2875–2880. doi:10.1002/cncr.30558 28387926 PMC5573914

