# Supplementary material for: A screen to identify antifungal antagonists reveals a variety of pharmacotherapies that induce echinocandin tolerance in Candida albicans
Source: Antimicrob Agents Chemother. 2025 Aug 18;69(10):e00484-25. doi: 10.1128/aac.00484-25 (PMC12486808; doi:10.1128/aac.00484-25)
Supplement: Supplemental figures — Fig. S1 to S4. [file aac.00484-25-s0001.pdf]

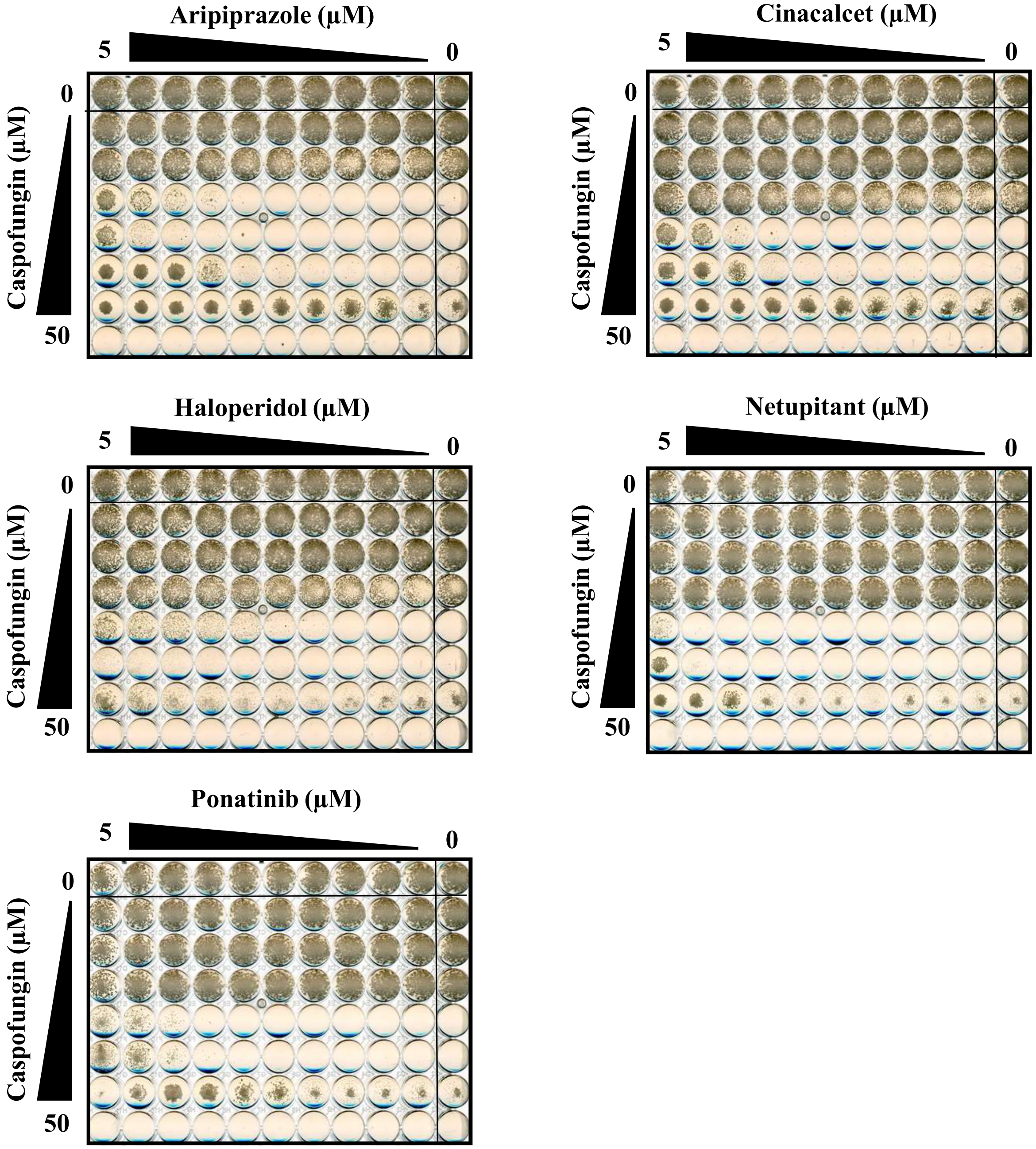

Figure S1. Echinocandin antagonists possess activity at sub-micromolar activity. Checkerboard assays were performed with *C. albicans* (SC5314) was grown in RPMI-pH 7 (2% glucose) with ***4-fold dilutions*** of caspofungin and ***2-fold dilutions*** of either aripiprazole, cinacalcet, haloperidol, netupitant, or ponatinib. Plates were incubated at 35°C and imaged after 72 hours. Images are representative of assays performed in biological duplicate.

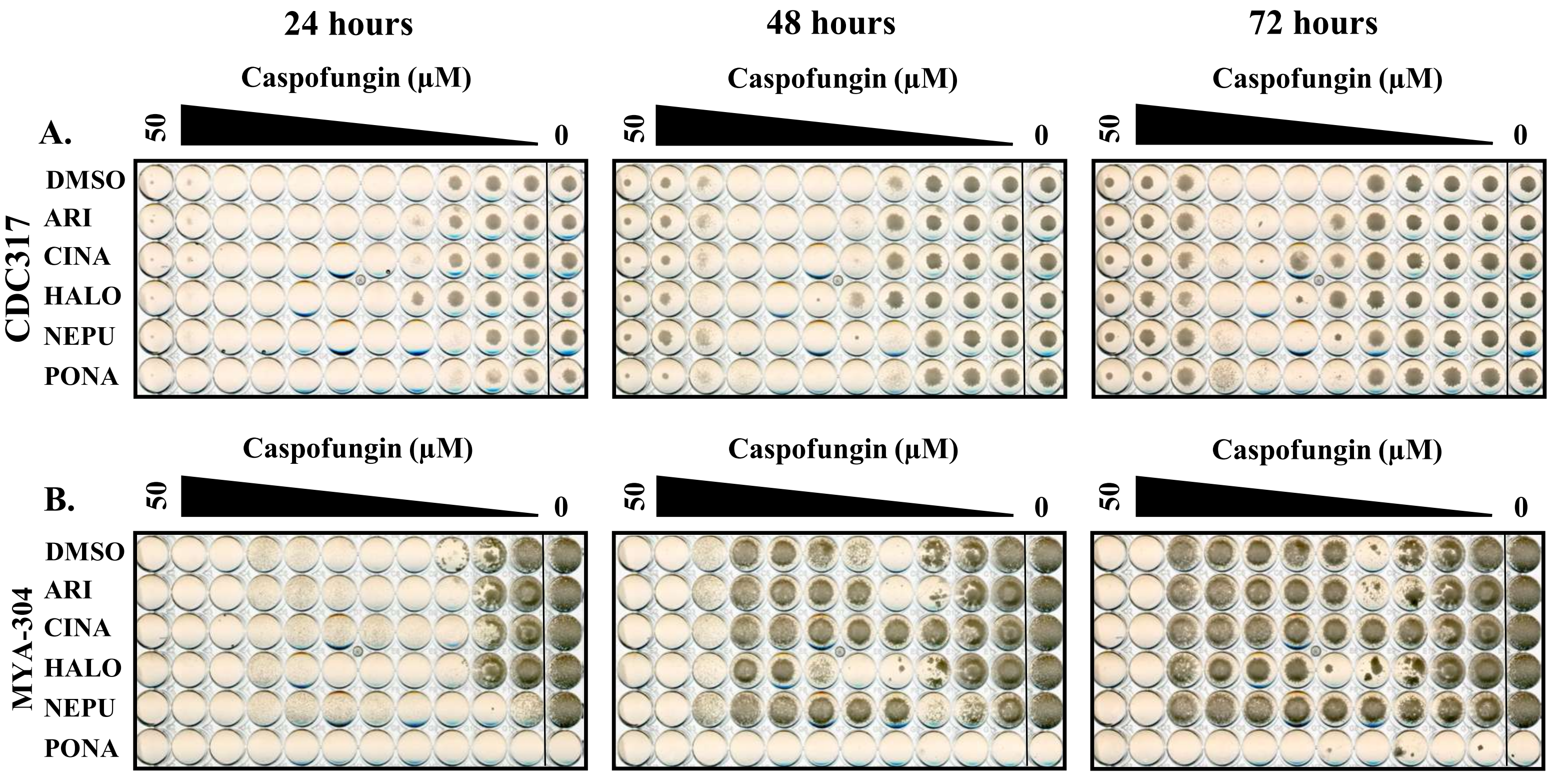

Figure S2. Selected antagonist activity is not species specific. *C. parapsilosis* strain CDC317 (**A**) or *C. tropicalis* strain MYA-304 (**B**) were grown in RPMI-pH 7 (2% glucose) supplemented with either 5 µM aripiprazole (ARI), 5 µM cinacalcet (CINA), 5 µM haloperidol (HALO), 5 µM netupitant (NEPU), 2.5 µM ponatinib (PONA), or vehicle (DMSO) in combination with increasing caspofungin concentrations. Plates were incubated at 35°C and imaged after 24, 48, and 72 hours. Images are representative of assays performed in biological duplicate.

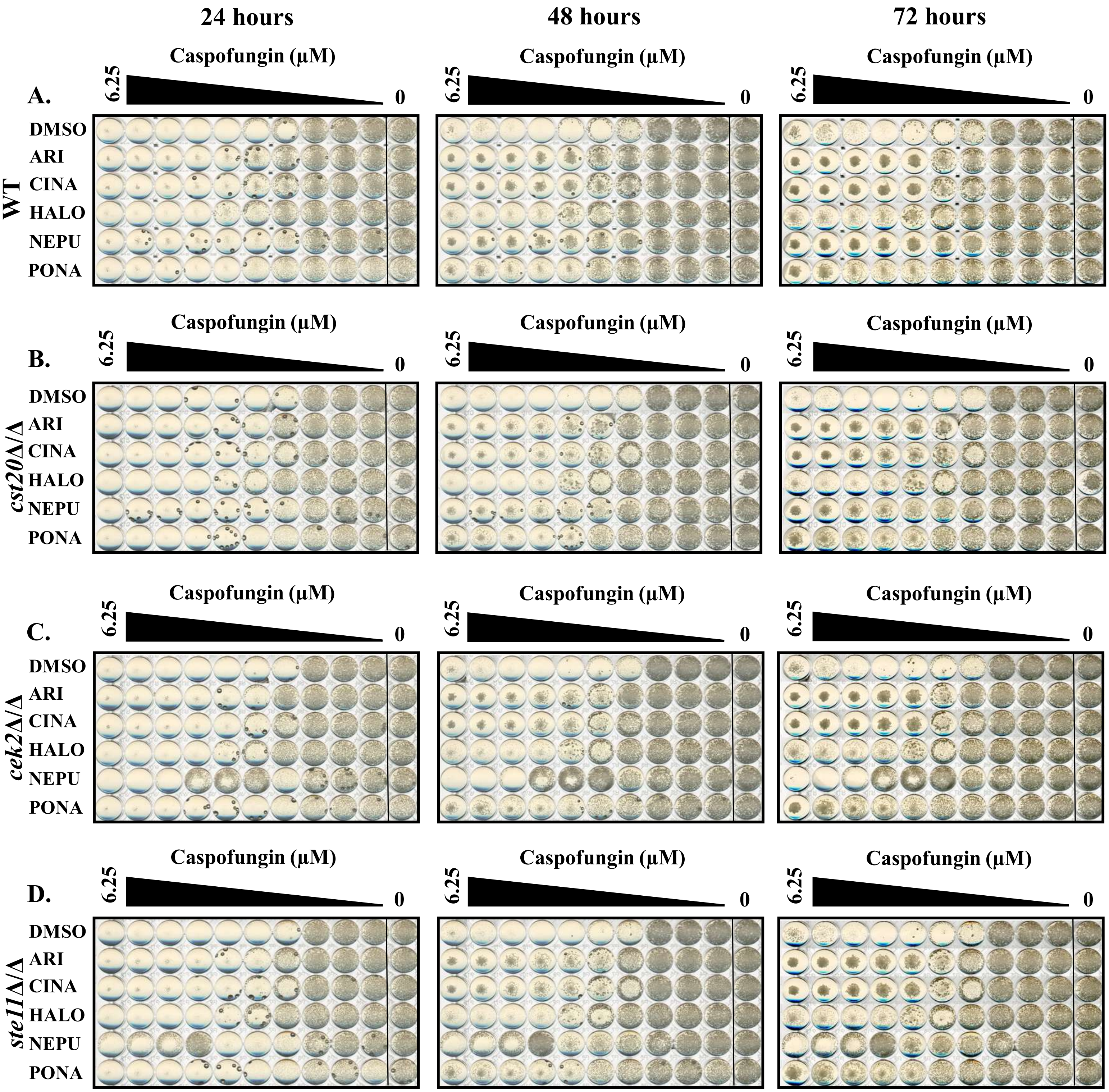

Figure S3. Echinocandin antagonist activity does not dependent upon components of Cek1p pathway. Caspofungin sensitivity of wild-type (SC5314 - **A**), *cst20Δ/Δ* (**B**), *cek2Δ/Δ* (**C**), and *ste11Δ/Δ* (**D**) *C. albicans* strains was compared in RPMI-pH 7 (2% glucose) supplemented with either 5 µM aripiprazole (ARI), 5 µM cinacalcet (CINA), 5 µM haloperidol (HALO), 5 µM netupitant (NEPU), 2.5 µM ponatinib (PONA), or vehicle (DMSO). Plates were incubated at 35°C, and imaged after 24, 48, and 72 hours. Images are representative of assays performed in biological duplicate.

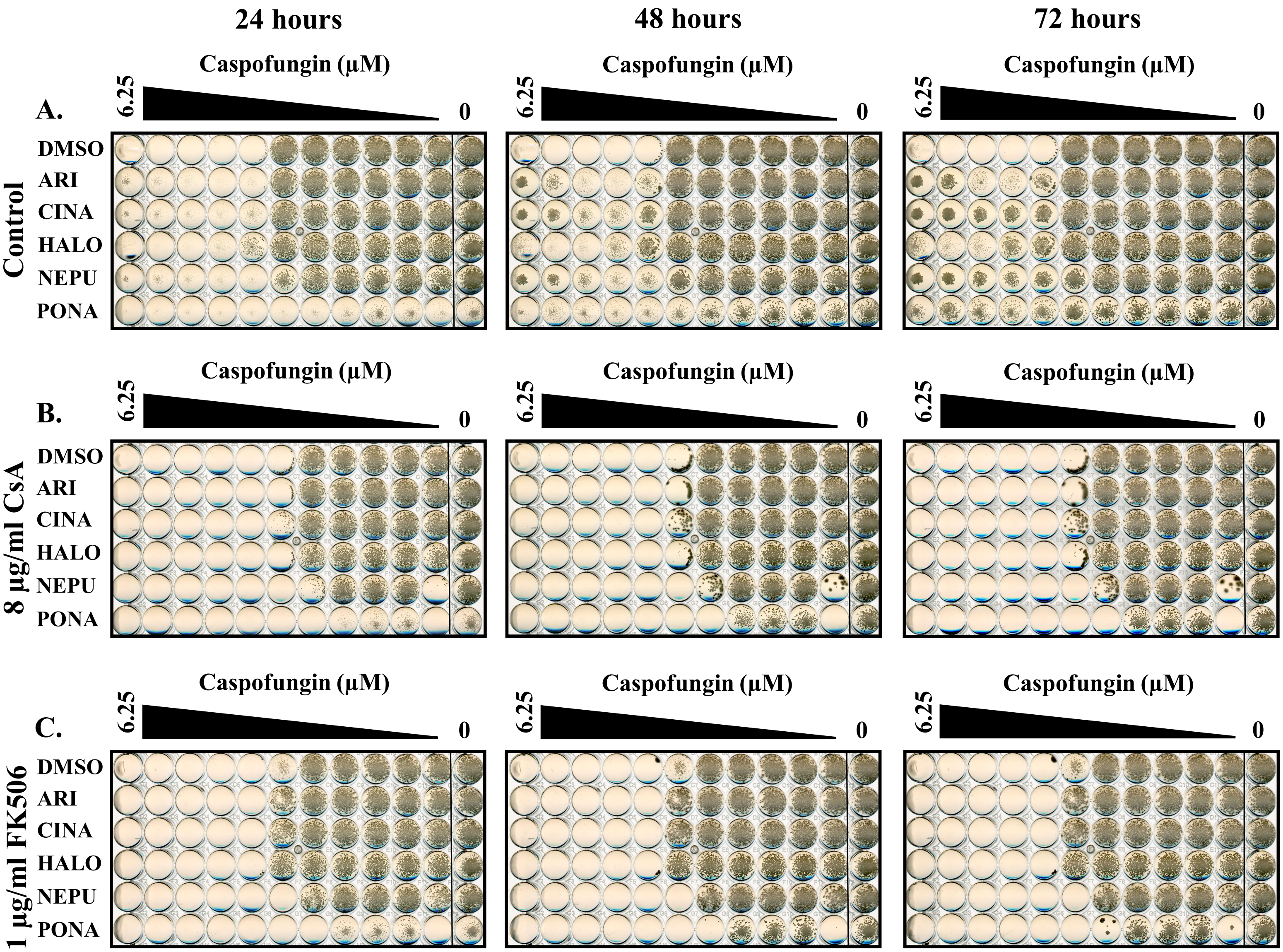

Figure S4. Inhibition of the calcineurin pathway suppress echinocandin antagonism. *C. albicans* SC5314 was grown in RPMI-pH 7 (2% glucose) supplemented with either 5 μM aripiprazole (ARI), 5 μM cinacalcet (CINA), 5 μM haloperidol (HALO), 5 μM netupitant (NEPU), 2.5 μM ponatinib (PONA), or vehicle (DMSO) in combination with increasing caspofungin concentrations. Medium was supplemented either with H_2O (vehicle control – **A**), 8 μg/ml Cyclosporin A (CsA - **B**), or 1 μg/ml FK506 (**C**). Plates were incubated at 35°C, and imaged after 24, 48, and 72 hours. Images are representative of assays performed in biological duplicate.
