# Supplementary material for: A screen to identify antifungal antagonists reveals a variety of pharmacotherapies that induce echinocandin tolerance in Candida albicans
Source: Antimicrob Agents Chemother. 2025 Aug 18;69(10):e00484-25. doi: 10.1128/aac.00484-25 (PMC12486808; doi:10.1128/aac.00484-25)
Supplement: Supplemental material — Fig. S5 to S8. [file aac.00484-25-s0002.pdf]

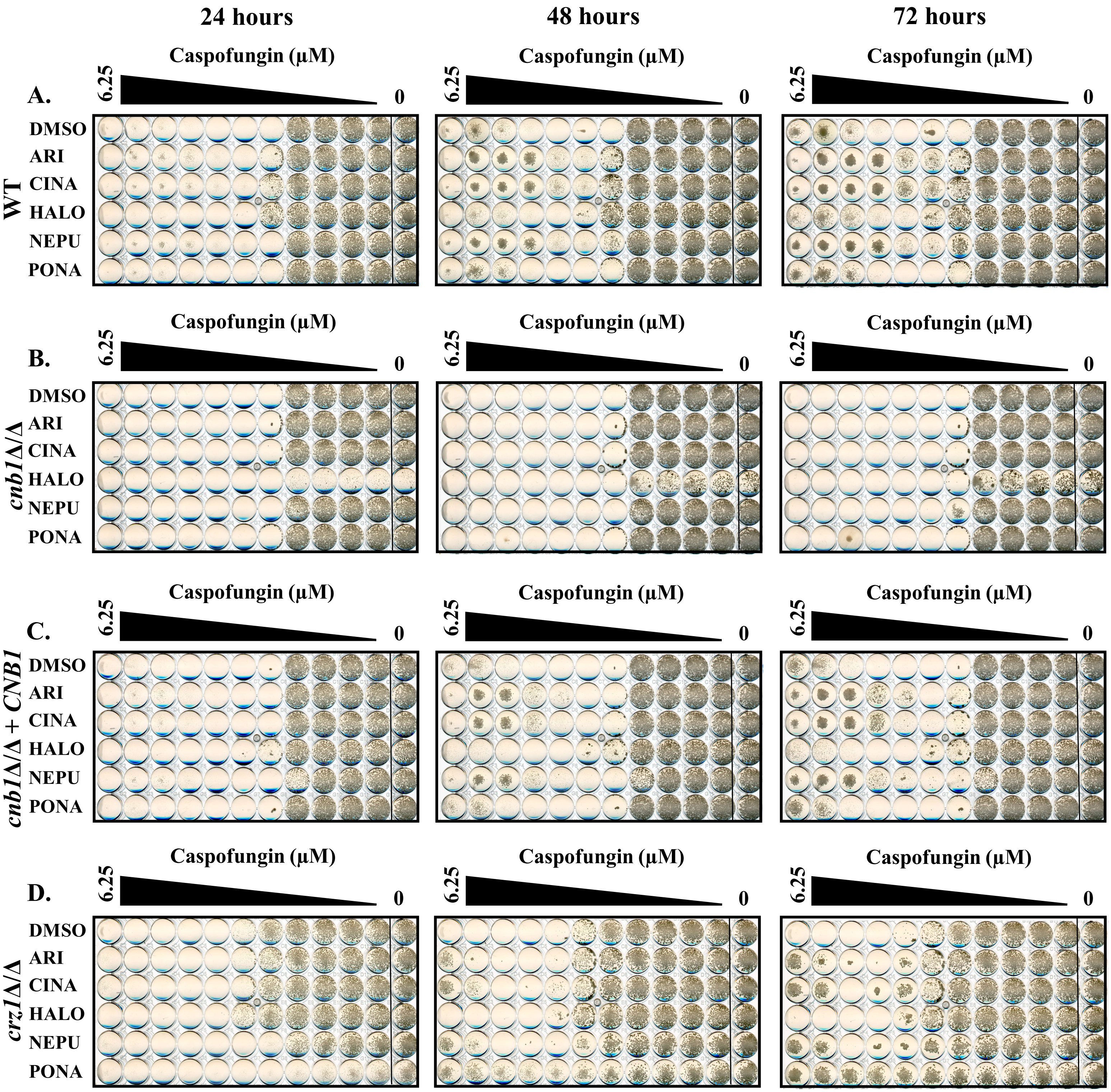

Figure S5. Echinocandin antagonism depends upon Cnb1p. The caspofungin sensitivity of wild-type (GP1 - **A**), *cnb1Δ/Δ* (**B**), *cnb1Δ/Δ* + *CNB1* (**C**), and *crz1Δ/Δ* (**D**) *C. albicans* strains was compared in RPMI-pH 7 (2% glucose) supplemented with either 5 µM aripiprazole (ARI), 5 µM cinacalcet (CINA), 5 µM haloperidol (HALO), 5 µM netupitant (NEPU), 2.5 µM ponatinib (PONA), or vehicle (DMSO). Plates were incubated at 35°C, and imaged after 24, 48, and 72 hours. Images are representative of assays performed in biological duplicate.

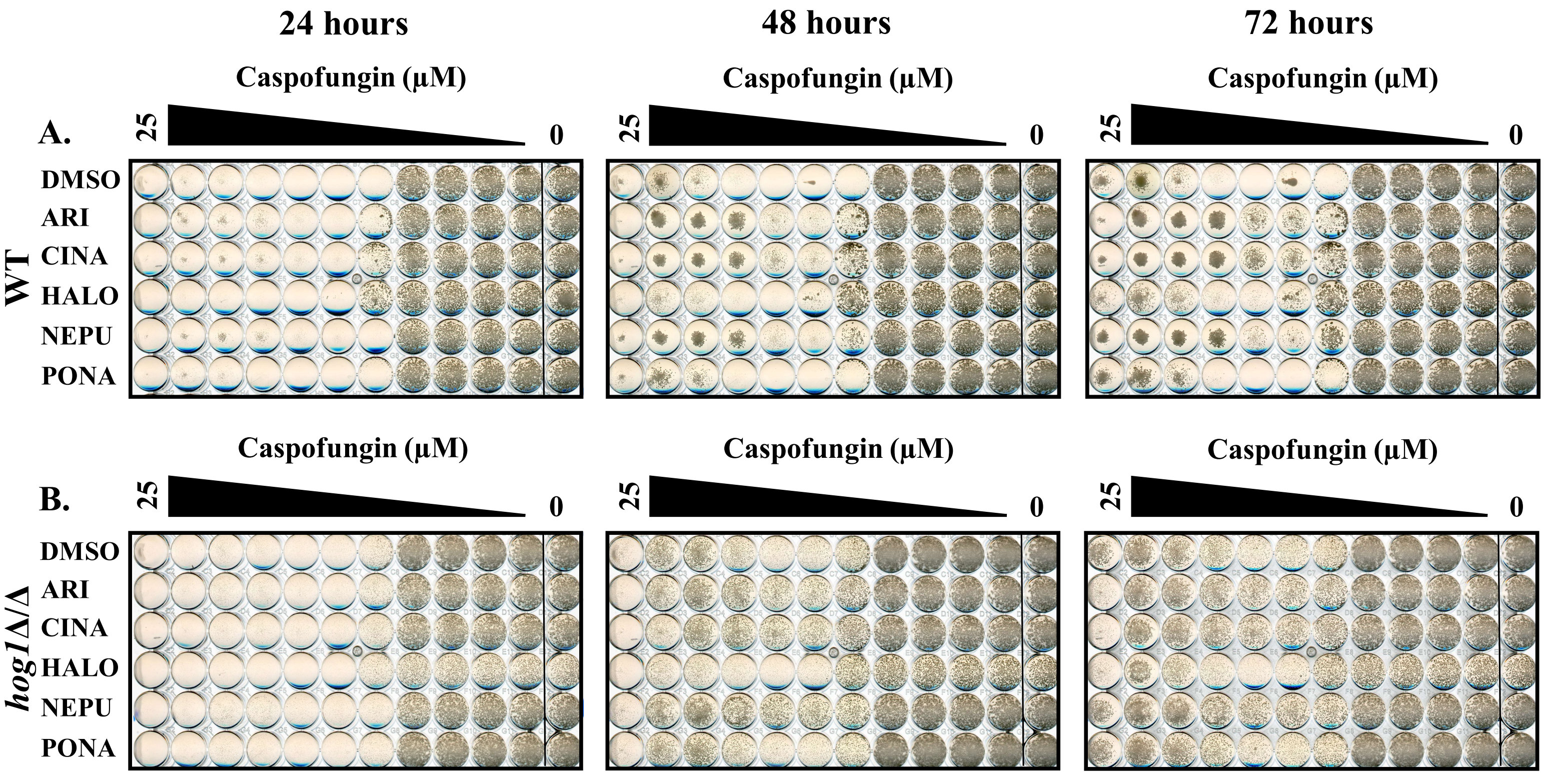

Figure S6. Deletion of *HOG1* induces echinocandin tolerance that is enhanced by antagonist treatment. *C. albicans* wild-type GP1 (**A**), and a *hog1Δ/Δ* mutant (**B**) were grown in RPMI-pH 7 (2% glucose) supplemented with either 5 µM aripiprazole (ARI), 5 µM cinacalcet (CINA), 5 µM haloperidol (HALO), 5 µM netupitant (NEPU), 2.5 µM ponatinib (PONA), or vehicle (DMSO) in combination with increasing caspofungin concentrations. Plates were incubated at 35°C, and imaged after 24, 48, and 72 hours. Images are representative of assays performed in biological duplicate.

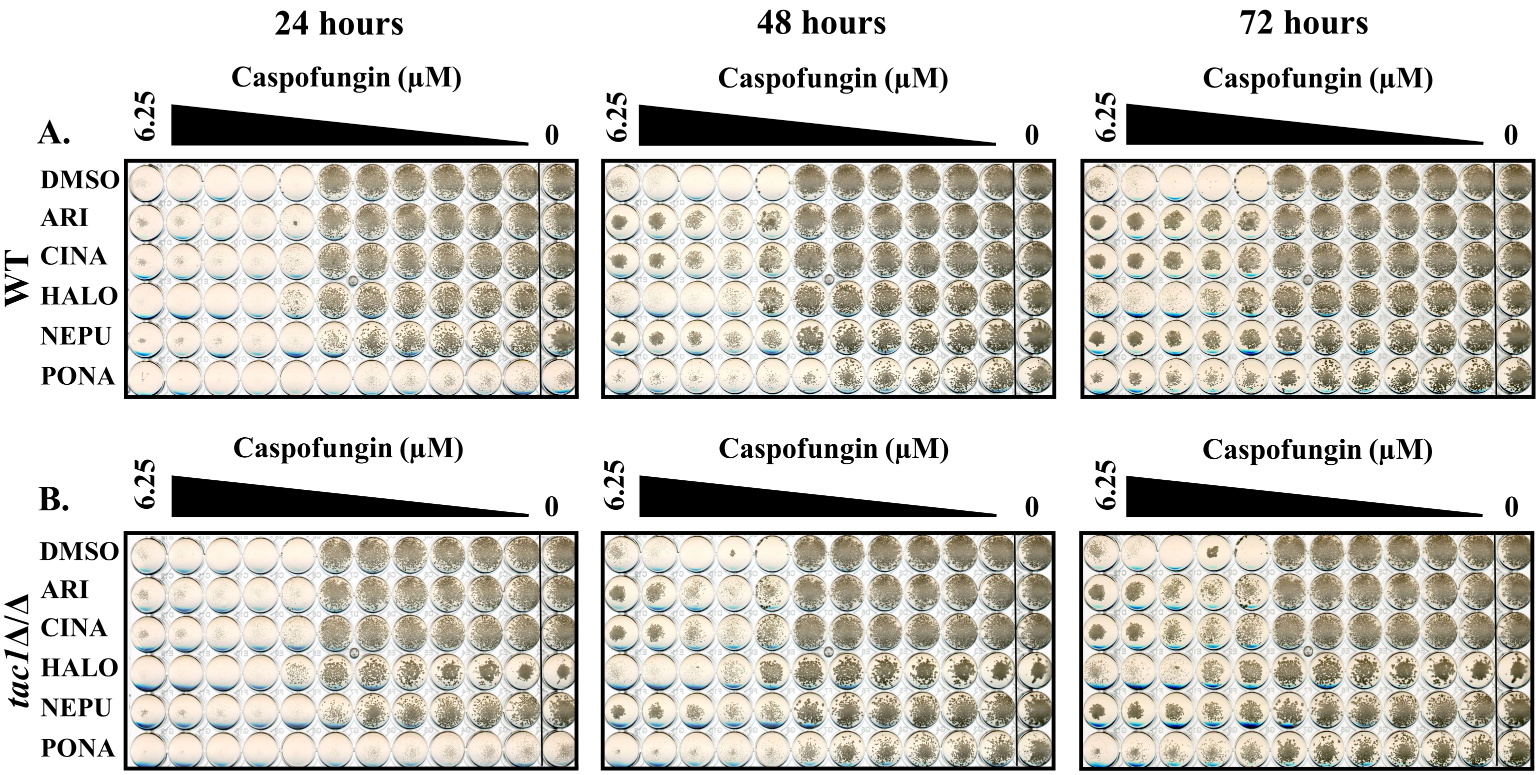

Figure S7. Echinocandin antagonism does not depend upon the Tac1p transcription factor. Wild-type GP1 (**A**), and *tac1Δ/Δ* (**B**) *C. albicans* strains were grown in RPMI-pH 7 (2% glucose) supplemented with either 5 μM aripiprazole (ARI), 5 μM cinacalcet (CINA), 5 μM haloperidol (HALO), 5 μM netupitant (NEPU), 2.5 μM ponatinib (PONA), or vehicle (DMSO) in combination with increasing caspofungin concentrations. Plates were incubated at 35°C, and imaged after 24, 48, and 72 hours. Images are representative of assays performed in biological duplicate.

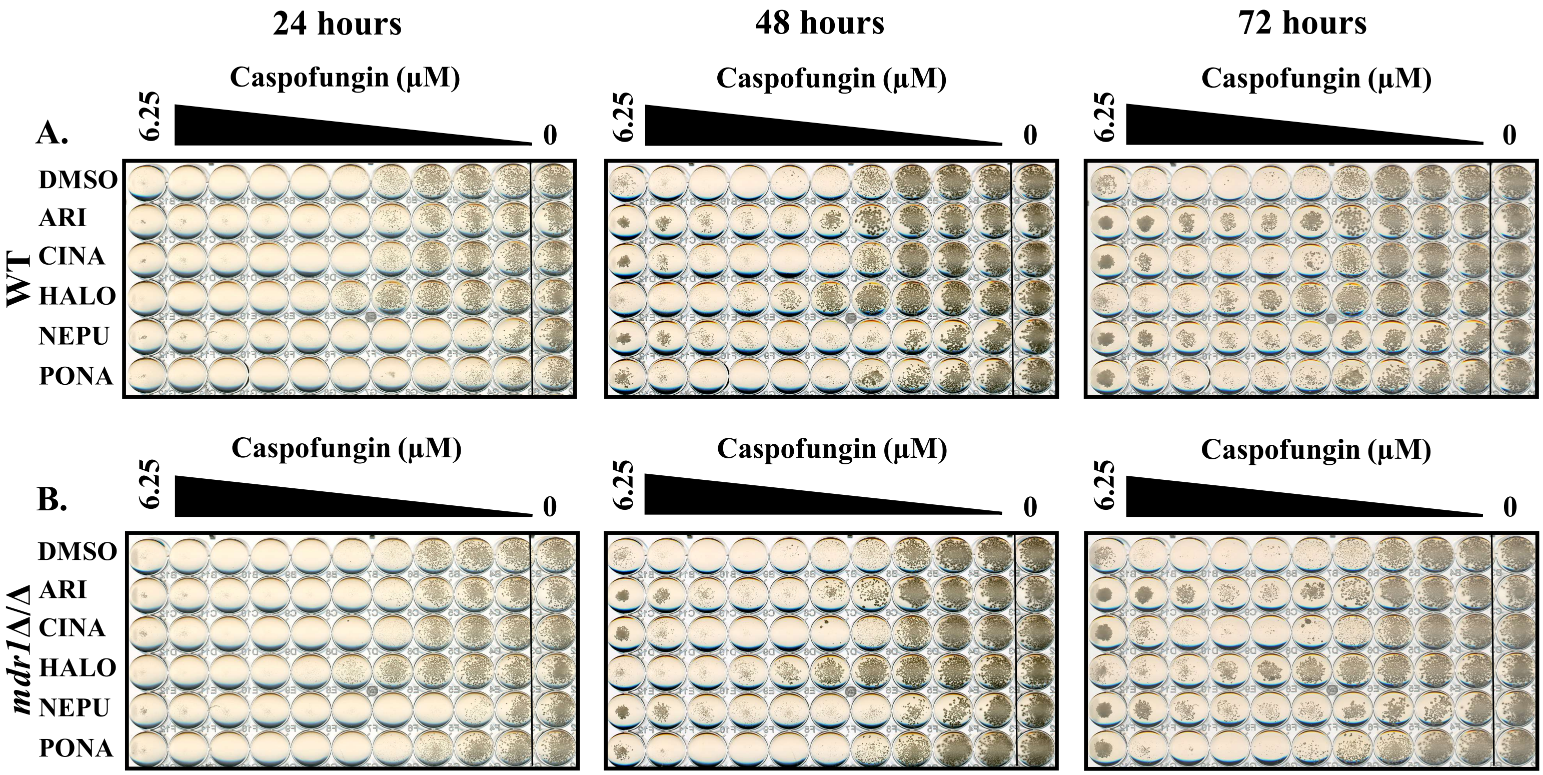

Figure S8. Echinocandin antagonism does not depend upon the Mdr1p efflux pump. The caspofungin sensitivity of wild-type (GP1 - **A**) and *mdr1Δ/Δ* (**B**) *C. albicans* strains was compared in RPMI-pH 7 (2% glucose) supplemented with either 5 µM aripiprazole (ARI), 5 µM cinacalcet (CINA), 5 µM haloperidol (HALO), 5 µM netupitant (NEPU), 2.5 µM ponatinib (PONA), or vehicle (DMSO). Plates were incubated at 35°C, and imaged after 24, 48, and 72 hours. Images are representative of assays performed in biological duplicate.
