# Supplementary material for: A screen to identify antifungal antagonists reveals a variety of pharmacotherapies that induce echinocandin tolerance in Candida albicans
Source: Antimicrob Agents Chemother. 2025 Aug 18;69(10):e00484-25. doi: 10.1128/aac.00484-25 (PMC12486808; doi:10.1128/aac.00484-25)
Supplement: Supplemental tables — Tables S1 to S3. [file aac.00484-25-s0003.pdf]

		Log$_2$ of Average Fold Change				
**Gene**	**Gene Description***	**Aripiprazole**	**Cinacalcet**	**Haloperidol**	**Netupitant**	**Ponatinib**
RTA3	7-transmembrane receptor protein involved in regulation of asymmetric lipid distribution in plasma membrane; involved in biofilm formation; putative drug-responsive regulatory site	3.423	3.706	1.818	4.198	2.059
WH11	White-phase yeast transcript; expression in opaques increases virulence/switching	2.809	3.507	1.984	3.878	2.661
PGA26	GPI-anchored adhesin-like protein of the cell wall; role in cell wall	-2.107	-2.912	-3.453	-2.580	-2.667
ALS3	Cell wall adhesin; epithelial adhesion, endothelial invasion	-2.545	-4.114	-4.510	-4.839	-1.146
SOU2	Protein similar to Sou1p	3.336	3.436	3.420	2.476	NA
orf19.6502	Short-chain dehydrogenase/reductase; upregulation correlates with clinical development of fluconazole resistance	2.249	2.483	1.961	2.382	NA
RSN1	Protein of unknown function	1.684	1.663	2.670	1.465	NA
GCY1	Aldo/keto reductase; mutation confers hypersensitivity to toxic ergosterol analog; farnesol-repressed; stationary phase enriched protein; flow model biofilm induced; Spider biofilm repressed	1.436	1.419	1.719	1.389	NA
LTV1	Putative GSE complex component; repressed by prostaglandins	-1.660	-1.542	-1.349	-1.237	NA
orf19.5710	Nucleoporin component of central core of the nuclear pore complex; mRNA binds She3p	-1.232	-1.617	-1.369	-1.737	NA
SEO1	Protein with similarity to permeases	-1.889	-2.155	-1.872	-2.312	NA
orf19.3475	Described as a Gag-related protein; hyphal induced; downregulation correlates with clinical development of fluconazole resistance	-1.724	-1.847	-3.245	-1.588	NA
DEF1	RNA polymerase II regulator; role in filamentation, epithelial cell escape, dissemination in RHE model; induced by fluconazole, high cell density; Efg1p/hyphal regulated; role in adhesion	-2.213	-2.071	-1.843	-2.361	NA
UME6	Zn(II)2Cys6 transcription factor; has a long 5'-UTR that regulates translational efficiency and controls transition to filamentous growth	-2.695	-3.967	-2.154	-4.213	NA
IHD1	GPI-anchored protein; alkaline, hypha-induced; regulated by Nrg1p, Rfg1p, Tup1p and Tsa1p	-3.511	-5.346	-2.929	-4.296	NA
ECE1	Candidalysin, cytolytic peptide toxin essential for mucosal infection; assembles into polymers that form pores in epithelial cells; hypha-specific protein	-3.211	-5.788	-2.523	-5.915	NA
HWP1	Hyphal cell wall protein; host transglutaminase substrate; opaque-, a-specific, alpha-factor induced	-3.118	-5.969	-2.933	-5.876	NA
CFL11	Superoxide-generating NADPH oxidase, produces extracellular burst of reactive oxygen species at growing cell tips during hyphal morphogenesis; regulated by Cdc42p	-4.302	-4.847	-4.012	-4.788	NA
CDR2	Multidrug transporter, ATP-binding cassette (ABC) superfamily; transports phospholipids, in-to-out direction; overexpressed in azole-resistant isolates	5.776	6.432	NA	6.186	4.105
orf19.4886	Putative adhesin-like protein	2.527	3.187	NA	2.959	2.313
OYE23	Putative NAPDH dehydrogenase	1.828	2.208	NA	2.751	2.596
ASR2	Adenylyl cyclase and stress responsive protein; induced in cyr1p or ras1p mutant	2.287	2.772	NA	3.218	1.074
orf19.4216	Putative heat shock protein; decreased expression in hyphae; transcription is increased in populations of cells exposed to fluconazole over multiple generations; overexpression increases resistance to farnesol and azoles	2.077	2.556	NA	2.254	1.857
orf19.86	Putative glutathione peroxidase; induced by peroxide, exposure to neutrophils and macrophage blood fractions	2.034	2.093	NA	2.503	1.654
HSP12	Heat-shock protein; induced by osmotic/oxidative/cadmium stress, fluphenazine treatment, low iron, *CDR1* and *CDR2* overexpression, or ssn6 or ssk1 null mutation; overexpression increases resistance to farnesol and azoles	1.881	2.355	NA	2.131	1.565
orf19.4907	Putative protein of unknown function; possibly transcriptionally regulated by Tac1p	1.927	1.987	NA	2.438	1.227
IFE1	Putative medium-chain alcohol dehydrogenase; rat catheter and Spider biofilm repressed	1.498	1.855	NA	2.240	1.880
SOD5	Cu-containing superoxide dismutase; protects against oxidative stress	-1.790	-2.456	NA	-2.170	-2.946
orf19.6200	Pry family pathogenesis-related protein	-3.348	-3.614	NA	-4.799	-3.497
orf19.3406	Predicted chloride transporter; member of conserved Mcm1p regulon	NA	-1.193	-1.382	-1.206	-1.811
PHO89	Putative phosphate permease	NA	-2.685	-2.514	-2.941	-1.850
GIT1	Glycerophosphoinositol permease; involved in utilization of glycerophosphoinositol as a phosphate source	NA	-2.433	-3.220	-2.046	-2.393

Table S1. *Candida albicans* gene transcripts responsive to four or more echinocandin antagonists. *as described in Candida Genome Database. NA = non applicable i.e. no significant change in transcript abundance compared to vehicle treated cells.

Drug Name	Cmax (μM)	Range Conc (μM)	Minimum Antagonistic Conc (μM)	Source
Aripiprazole	0.76-1.008*	0.22-0.78	0.625	Micromedex
Cinacalcet HCl	0.018	0.018-0.143	1.25	Micromedex
Haloperidol	0.007	0.002-0.027	0.313	Micromedex, 1
Netupitant	0.750	NA	5	Micromedex
Ponatinib	0.140	0.056	1.25	Micromedex, 2

Table S2. Activity of select antagonists at therapeutically relevant concentrations compared *in vitro* activity.
* Aripiprazole concentrations vary based on oral vs IM dosing.

References

1. **Uematsu T, Matsuno H, Sato H, Hirayama H, Hasegawa K, Nakashima M.** Steady-state pharmacokinetics of haloperidol and reduced haloperidol in schizophrenic patients: analysis of factors determining their concentrations in hair. J Pharm Sci. 1992 Oct;81(10):1008-11. doi: 10.1002/jps.2600811010. PMID: 1432610.
2. **Nicolini FE, Basak GW, Kim DW, Olavarria E, Pinilla-Ibarz J, Apperley JF, Hughes T, Niederwieser D, Mauro MJ, Chuah C, Hochhaus A, Martinelli G, DerSarkissian M, Duh MS, McGarry LJ, Kantarjian HM, Cortes JE.** Overall survival with ponatinib versus allogeneic stem cell transplantation in Philadelphia chromosome-positive leukemias with the T315I mutation. Cancer. 2017 Aug 1;123(15):2875-2880. doi: 10.1002/cncr.30558. Epub 2017 Apr 7. PMID: 28387926; PMCID: PMC5573914.

Strain	Genotype	Reference
SC5314	*C. albicans* reference strain	1
GP1	*C. albicans arg4*Δ/Δ::*ARG4 his1*Δ/Δ::*HIS1 ura3*Δ/Δ::*URA3*	2
CAI4+pKE1-NLUC	*C. albicans ura3*Δ/Δ:*URA3*:ACT1prNLUC	3
TW1	*C. albicans* clinical isolate	4
ATT10231	*C. albicans* reference strain	5
JKC19	*C. albicans ura3*Δ::*imm434*/*ura3*Δ::*imm434 cph1*::*hisG*/*cph1*::*hisG-URA3-hisG*	6
HLC52	*C. albicans ura3*Δ::*imm434*/*ura3*Δ::*imm434 efg1*::*hisG*/*efg1*::*hisG-URA3-hisG*	6
HLC54	*C. albicans ura3*Δ::*imm434*/*ura3*Δ::*imm434 cph1*::*hisG*/*cph1*::*hisG efg1*::*hisG*/*efg1*::*hisG-URA3-hisG*	6
473	*C. albicans* AWY307 *mkc1*ΔΔ *CHS3/CHS3-GFP LEU2/leu2*Δ	7
323	*C. albicans* AWY208 *cek1*ΔΔ *LEU2/leu2*Δ	8
JRB64	*C. albicans ura3*Δ::Δ*imm434*/*ura3*Δ::Δ*imm434 his1*::*hisG*::*HIS1*/*his1*::*hisG arg4*::*hisG*/*arg4*::*hisG*::*cnb1*::*UAU*/*cnb1*::*ARG4*	9
MCC85	*C. albicans ura3*Δ::Δ*imm434*/*ura3*Δ::Δ*imm434 his1*::*hisG*::*CNB1-HIS1*/*his1*::*hisG arg4*::*hisG*/*arg4*::*hisG*::*cnb1*::*UAU*/*cnb1*::*ARG4*	10
OCC1.1	*C. albicans crz1*Δ/Δ	11
MMM50	*C. albicans hog1*Δ/Δ *LEU2/leu2*Δ	8
CDC317	*C. parapsilosis* reference strain	12
MYA-304	*C. tropicalis* reference strain	13
MA3L-1	*C. albicans his1*Δ/Δ *arg4*Δ/Δ *mdr1*Δ::*HIS1*/*mdr1*Δ::*ARG4 ura3*Δ/Δ:*URA3*	14

Table S3. List of strains used in study.

References.

1. **Odds FC, Brown AJ, Gow NA.** *Candida albicans* genome sequence: a platform for genomics in the absence of genetics. Genome Biol. 2004;5(7):230. doi: 10.1186/gb-2004-5-7-230. Epub 2004 Jun 11. PMID: 15239821; PMCID: PMC463275.
2. **Willems HME, Lowes DJ, Barker KS, Palmer GE, Peters BM.** Comparative Analysis of the Capacity of the *Candida* Species To Elicit Vaginal Immunopathology. Infect Immun. 2018 Nov 20;86(12):e00527-18. doi: 10.1128/IAI.00527-18. PMID: 30249743; PMCID: PMC6246903.
3. **Luna-Tapia A, Tournu H, Peters TL, Palmer GE**. Endosomal Trafficking Defects Can Induce Calcium-Dependent Azole Tolerance in *Candida albicans*. Antimicrob Agents Chemother. 2016 Nov 21;60(12):7170-7177. doi: 10.1128/AAC.01034-16. PMID: 27645241; PMCID: PMC5118996.
4. **White TC.** Increased mRNA levels of ERG16, CDR, and MDR1 correlate with increases in azole resistance in *Candida albicans* isolates from a patient infected with human immunodeficiency virus. Antimicrob Agents Chemother. 1997 Jul;41(7):1482-7. doi: 10.1128/AAC.41.7.1482. PMID: 9210670; PMCID: PMC163944.
5. **Zore G, Abdulghani M, Kodgire S, Kazi R, Shelar A, Patil R.** Proteome dataset of *Candida albicans* (ATCC10231) opaque cell. BMC Res Notes. 2024 Jan 2;17(1):2. doi: 10.1186/s13104-023-06661-z. PMID: 38167002; PMCID: PMC10759580.
6. **Peters BM, Palmer GE, Nash AK, Lilly EA, Fidel PL Jr, Noverr MC**. Fungal morphogenetic pathways are required for the hallmark inflammatory response during *Candida albicans* vaginitis. Infect Immun. 2014 Feb;82(2):532-43. doi: 10.1128/IAI.01417-13. Epub 2013 Nov 11. PMID: 24478069; PMCID: PMC3911367.
7. **Wagner AS, Lumsdaine SW, Mangrum MM, Reynolds TB**. Caspofungin-induced β(1,3)-glucan exposure in *Candida albicans* is driven by increased chitin levels. mBio. 2023 Aug 31;14(4):e0007423. doi: 10.1128/mbio.00074-23. Epub 2023 Jun 28. PMID: 37377417; PMCID: PMC10470516.
8. **Chen T, Jackson JW, Tams RN, Davis SE, Sparer TE, Reynolds TB**. Exposure of *Candida albicans* β (1,3)-glucan is promoted by activation of the Cek1 pathway. PLoS Genet. 2019 Jan 31;15(1):e1007892. doi: 10.1371/journal.pgen.1007892. PMID: 30703081; PMCID: PMC6372213.
9. **Blankenship JR, Wormley FL, Boyce MK, Schell WA, Filler SG, Perfect JR, Heitman J.** Calcineurin is essential for *Candida albicans* survival in serum and virulence. Eukaryot Cell. 2003 Jun;2(3):422-30. doi: 10.1128/EC.2.3.422-430.2003. PMID: 12796287; PMCID: PMC161442.
10. **Cruz MC, Goldstein AL, Blankenship JR, Del Poeta M, Davis D, Cardenas ME, Perfect JR, McCusker JH, Heitman J.** Calcineurin is essential for survival during membrane stress in *Candida albicans*. EMBO J. 2002 Feb 15;21(4):546-59. doi: 10.1093/emboj/21.4.546. PMID: 11847103; PMCID: PMC125859.
11. **Reynolds TB, Hopkins BD, Lyons MR, Graham TR.** The high osmolarity glycerol response (HOG) MAP kinase pathway controls localization of a yeast golgi glycosyltransferase. J Cell Biol. 1998 Nov 16;143(4):935-46. doi: 10.1083/jcb.143.4.935. PMID: 9817752; PMCID: PMC2132948.
12. **Bergin S, Doorley LA, Rybak JM, Wolfe KH, Butler G, Cuomo CA, Rogers PD.** 2024. Analysis of clinical *Candida parapsilosis* isolates reveals copy number variation in key fluconazole resistance genes. Antimicrob Agents Chemother 68:e01619-23. https://doi.org/10.1128/aac.01619-23
13. **Chen YC, Lo HJ, Chiang HS.** *Candida tropicalis*-derived vitamin B3 exerts protective effects against intestinal inflammation by promoting IL-17A/IL-22-dependent epithelial barrier function. Gut Microbes. 2024 Jan-Dec;16(1):2416922. doi: 10.1080/19490976.2024.2416922. Epub 2024 Oct 27. PMID: 39462273; PMCID: PMC11524206.
14. **Luna-Tapia A, Kerns ME, Eberle KE, Jursic BS, Palmer GE.** Trafficking through the late endosome significantly impacts Candida albicans tolerance of the azole antifungals. Antimicrob Agents Chemother. 2015 Apr;59(4):2410-20. doi: 10.1128/AAC.04239-14. Epub 2015 Feb 9. PMID: 25666149; PMCID: PMC4356793.
